# Prediction of Mutational Tolerance in HIV-1 Protease and Reverse Transcriptase Using Flexible Backbone Protein Design

**DOI:** 10.1371/journal.pcbi.1002639

**Published:** 2012-08-23

**Authors:** Elisabeth Humphris-Narayanan, Eyal Akiva, Rocco Varela, Shane Ó Conchúir, Tanja Kortemme

**Affiliations:** 1 Graduate Group in Biophysics, University of California, San Francisco, San Francisco, California, United States of America; 2 California Institute for Quantitative Biomedical Research, University of California, San Francisco, San Francisco, California, United States of America; 3 Department of Bioengineering and Therapeutic Sciences, University of California, San Francisco, San Francisco, California, United States of America; 4 Graduate Program in Bioinformatics, University of California, San Francisco, San Francisco, California, United States of America; Weizmann Institute of Science, Israel

## Abstract

Predicting which mutations proteins tolerate while maintaining their structure and function has important applications for modeling fundamental properties of proteins and their evolution; it also drives progress in protein design. Here we develop a computational model to predict the tolerated sequence space of HIV-1 protease reachable by single mutations. We assess the model by comparison to the observed variability in more than 50,000 HIV-1 protease sequences, one of the most comprehensive datasets on tolerated sequence space. We then extend the model to a second protein, reverse transcriptase. The model integrates multiple structural and functional constraints acting on a protein and uses ensembles of protein conformations. We find the model correctly captures a considerable fraction of protease and reverse-transcriptase mutational tolerance and shows comparable accuracy using either experimentally determined or computationally generated structural ensembles. Predictions of tolerated sequence space afforded by the model provide insights into stability-function tradeoffs in the emergence of resistance mutations and into strengths and limitations of the computational model.

## Author Summary

Many related protein sequences can be consistent with the structure and function of a given protein, suggesting that proteins may be quite robust to mutations. This tolerance to mutations is frequently exploited by pathogens. In particular, pathogens can rapidly evolve mutated proteins that have a new function - resistance against a therapeutic inhibitor - without abandoning other functions essential for the pathogen. This principle may also hold more generally: Proteins tolerant to mutational changes can more easily acquire new functions while maintaining their existing properties. The ability to predict the tolerance of proteins to mutation could thus help both to analyze the emergence of resistance mutations in pathogens and to engineer proteins with new functions. Here we develop a computational model to predict protein mutational tolerance towards point mutations accessible by single nucleotide changes, and validate it using two important pathogenic proteins and therapeutic targets: the protease and reverse transcriptase from HIV-1. The model provides insights into how resistance emerges and makes testable predictions on mutations that have not been seen yet. Similar models of mutational tolerance should be useful for characterizing and reengineering the functions of other proteins for which a three-dimensional structure is available.

## Introduction

The relationship between protein sequence and structure is fundamental for protein function, evolution and design [1], [2]. Many sequences are compatible with a given structure and function and thus proteins are often robust to point mutation [3], [4], [5]. The concept of “tolerated sequence space" - the set of sequences that accommodate a given structure and function - has been applied to characterize the emergence of protein families [6], to describe protein interaction specificity [7] and to explain the evolution of new protein functions [8], [9].

Tolerated sequence variability (robustness to mutation) should be an advantage if proteins need to satisfy multiple functional constraints simultaneously. If each constraint can be accommodated by many sequences, it should be easier to find a subset of sequences that satisfy multiple requirements [10]. Moreover, a protein that has many tolerated sequences may be able to accommodate new constraints without abandoning some existing function [8], [11], [12].

An example of this ability of proteins to rapidly adapt to new pressures is the emergence of drug-resistance mutations in pathogens. In many cases, variants of pathogenic proteins that are resistant to inhibitors appear quickly, while still preserving their essential functions for the pathogen. It is likely that some of these mutations are already present in the population as part of naturally occurring nearly neutral sequence variation [13] and are then selected by inhibitor treatment. Thus, the *a priori* prediction of the tolerated sequence variation of pathogenic proteins would have implications for development of inhibitors against which resistance is less likely to arise quickly [14].

Here we develop and assess a computational approach to predict the tolerated space of single mutations around a given protein sequence. As model systems for validating our approach, we use the protease and reverse transcriptase from HIV-1. With more than 50,000 known sequences and several hundred experimentally determined structures, these two viral proteins are among the best-characterized systems available of tolerated variants around a native sequence. Because protein sequences have been collected before and after viral inhibitor treatment [15], predictions of mutational tolerance can be assessed in both a nearly neutral setting and under selective pressure to evolve resistance mutations. In testing our model for HIV-1 protease mutational tolerance, we also make use of a large-scale mutagenesis experiment which evaluated the *in vivo* function of roughly 50% of all mis-sense mutations reachable by a single-nucleotide change from a starting consensus sequence [16].

We find that our approach, which employs computational protein design methods in Rosetta [17], recapitulates a substantial fraction of mutations experimentally observed to be tolerated by HIV protease and reverse transcriptase. For accurate predictions, we show that it is critical to treat the protein not as a rigid single structure, but to allow conformational variation to accommodate sequence changes [18], [19], [20]. We show that essentially the same prediction accuracy is achieved when obtaining conformational variation from an ensemble of experimentally determined structures of HIV protease [21] or reverse transcriptase, or from computationally generated conformational ensembles [18], [19], [20], [22]. We thus expect our approach to also be applicable to systems for which there is only one structure known. Computational models of accessible mutational space, such as the one presented here, may prove generally useful for describing the evolvability of proteins by forecasting the emergence of mutations that can enable new protein functions [8].

## Results

### Description of the computational model: integrating functional constraints over multiple structures

To predict a protein's tolerance to mutation, ideally all constraints acting on that protein should be modeled explicitly. In addition, accurate predictions of mutational tolerance may require that conformational adjustments in response to mutation be considered. Here we present a methodology that incorporates multiple functional constraints as well as backbone flexibility into RosettaDesign [17] and apply it to the prediction of mutational tolerance. We first consider the viral protein HIV-1 protease, and later extend our results to HIV-1 reverse transcriptase.

HIV-1 protease is an ideal test system for several reasons. First, the mutational tolerance of HIV-1 protease is well characterized: mutations of HIV-1 protease, including those causing resistance to protease inhibitors in HIV treatment, have been extensively documented and are available in the Stanford HIV-1 Drug Resistance Database [15]. Second, HIV-1 protease is under at least three structural and functional constraints that are straightforward to model: (1) the 99-residue protease sequence must adopt a stable fold; (2) the active enzyme is a homodimer, and (3) the dimeric form must bind at least 10 endogenous peptides. Finally, HIV-1 protease is structurally well characterized, with hundreds of crystal structures of native and mutated forms in the apo state or with peptide or inhibitors bound.

**Figure 1:**
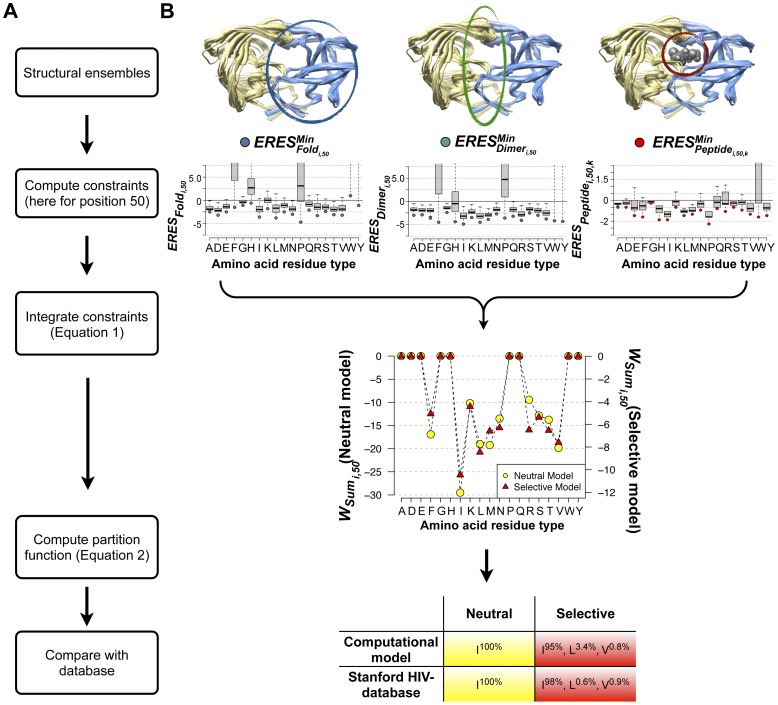
Computational model for predicting mutational tolerance. (**A**) Flowchart illustrating key steps. (**B**) Example calculations for position 50 in HIV-1 protease. For each position in the protein of interest, all amino acid residue types (except cysteine) are computationally modeled onto each structure in an ensemble of backbone structures. For each mutation, the Rosetta per-residue energy contribution (*ERES*) is recorded for each structure. These values are depicted as boxplots showing the variation in the *ERES* scores calculated over the ensemble (*ERES_Fold_* and *ERES_Dimer_* scores are shown for 263 experimentally determined protease structures; *ERES_Peptide_* scores are shown for 19 structures with a substrate peptide bound). Next, the minimum (*i.e.* most favorable) *ERES* score observed among all structures in the ensemble is determined with respect to fold stability (**left** boxplot, blue circles), dimer stability (**middle** boxplot, green circles) and binding to 10 substrate peptides (**right** boxplot, red circles). These minimum scores are then weighted and summed for each point mutation to yield *W_Sum_* for each position j and amino acid i (**Equation 1**). Sums are performed using either neutral or selective weights (see **Table S4**). 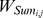 scores are combined using **Equation (2)** to give predicted frequencies for each residue type (superscript). For comparison, the mutational frequencies for position 50 observed in the Stanford HIV-1 database before and after inhibitor treatment are shown below the predicted frequencies (superscripts for each observed residue type).

**Figure 1A** outlines the computational strategy for predicting mutational tolerance, starting from three-dimensional structural information on the protein of interest. **Figure 1B** gives an example of the calculations for one sequence position in HIV-1 protease. We started from the consensus sequence for HIV-1 protease (see Methods), and considered all individual point mutations independently (the simplest model of mutational space around a given sequence). We used RosettaDesign [10], [17], [18] to mutate, *in silico*, each sequence position to 19 naturally occurring amino acid types (mutations to and from cysteine were excluded; see Methods). For each residue change, the side-chain conformations were optimized around the site of mutation. We then calculated the per-residue energy contribution (termed *ERES*) of each point mutation using the Rosetta all-atom force field (see Methods). *ERES* scores were computed with respect to the three functional pressures described above: (1) the stability of the protease fold (*ERES_Fold_*, **Figure 1B**, left); (2) the stability of the protease dimer interface (*ERES_Dimer_*, **Figure 1B**, middle); and (3) the stability of the binding interactions with endogenous substrate peptides (*ERES_Peptide_*, **Figure 1B**, right). The model has the following key steps and components (**Figure 1A**):

1. **Structural ensembles.** To take possible conformational adjustments in response to mutation into account, we incorporated structural flexibility into our model in two different ways. First, we calculated mutational tolerance using an ensemble of experimentally determined protease structures (**Figure 1** and **Table S1**). For each member of the ensemble, all bound peptides and inhibitors were removed and mutations originally present in the structure were computationally reverted back to the consensus sequence (Methods). RosettaDesign was used to model the sequence reversion onto the fixed backbone of each ensemble member by performing a side-chain repacking step around the sites of the residue changes, followed by side-chain torsion minimization to reduce potential steric clashes. Alternatively, to test for possible bias of this protocol towards mutations already present in some of the experimentally determined structures, we also performed a second analysis where we computationally generated ensembles of backbones starting from a single experimentally determined structure that had few or no mutations (described below).
2. **Compute constraints.** For both experimentally determined and computationally generated structural ensembles, *ERES_Fold_* and *ERES_Dimer_* were computed independently for each ensemble member. To estimate *ERES_Peptide_* for interaction with protease substrates, we used a second set of 19 crystallographic structures and structural models of HIV-1 protease bound to 10 known peptide cleavage substrates (**Table S2**). There was often considerable variation in *ERES_Fold_*, *ERES_Dimer_* and *ERES_Peptide_* scores calculated from different structures in the ensembles (**Figure 1B** **)**. Thus, to obtain a single *ERES* score for each constraint (Fold, Dimer and Peptide) and mutation, we selected the most favorable (*e.g.* lowest) predicted *ERES* score (denoted *ERES^Min^*) for a given substitution modeled on all ensemble members (circles, **Figure 1B**). This method effectively selects the backbone conformation best accommodating each substitution.
3. **Integrate constraints.** We integrated the effects of each mutation on the different modeled constraints by computing a weighted sum (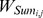) of the estimated *ERES^Min^* contributions of each amino acid substitution, i, to fold stability, dimer stability and peptide binding for every protein sequence position, j (**Equation 1**):
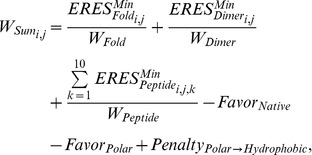
(1)where *1≤i≤19* is the index of a given amino acid type at a position and *1≤j≤n* is the index of the sequence position considered. 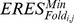 and 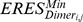 denote the best fold and dimer stability *ERES* scores computed for amino acid *i* at protein position *j*. 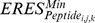 stands for the best *ERES* contribution towards substrate binding computed for amino acid *i* at protease position *j* with peptide *k*. *W_Fold_*, *W_Dimer_* and *W_Peptide_* represent weights for each constraint modeled. The constant *Favor_Native_* favors the native residue type at each position; this term effectively sets an overall mutation frequency, and can be parameterized to fit observed frequencies (in this case in the Stanford database). To model selection pressure for solubility, constant components favor polar residues (*Favor_Polar_*) and disfavor substitutions of polar residues with hydrophobic residues (*Penalty_Polar→Hydrophobic_*). 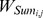 was set to zero for all mutations that were not accessible by a single nucleotide change from the consensus sequence (**Table S3**). This process highly disfavored, but did not forbid, point mutations that are less likely to be sampled at the nucleotide level (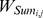
is expected to be negative (favorable) for many amino acids).
  Using **Equation 1**, we first created a “nearly neutral" model of mutational tolerance by weighting the three constraints (*W_Fold_*, *W_Dimer_* and *W_Peptide_*) approximately equally (Methods, **Table S4**). Second, we parameterized the model to create “selective" settings that simulate pressure under inhibitor treatment. This selective model allows accumulation of mutations near the dimer interface and the active site by down-weighting the *ERES_Dimer_* and *ERES_Peptide_* contributions. Model parameter values for **Equation 1** are given in **Table S4**.
4. **Compute partition function to predict mutational frequencies.** To obtain predicted frequencies for each amino acid *i* at each position *j* (*P_i,j_*), we computed a partition function (**Equation 2**):
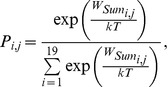
(2)where 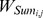 for amino acid *i* at position *j* is as in Eq.(1) and *kT* = 0.6 kcal/mol.
5. **Compare with database.** Finally, we assessed the model by comparing the predicted mutational frequencies to those experimentally observed. We used overall position-dependent mutational frequencies (by adding the mutational frequencies of non-native amino acids predicted at each position) and also asked whether the model recapitulates mutations to individual amino acids residue types at each position. In total, we analyzed mutational tolerance for 96/99 residues of HIV protease (for excluded residues see Methods).

### Overall model performance

Evaluating the robustness of a protein to mutation requires accurate distinction between sites that display amino acid variation and ones that do not. Some protein sites are mutation intolerant under neutral conditions but become more tolerant under selective pressure; other sites are intolerant to mutation under both neutral and selective conditions.

**Figure 2:**
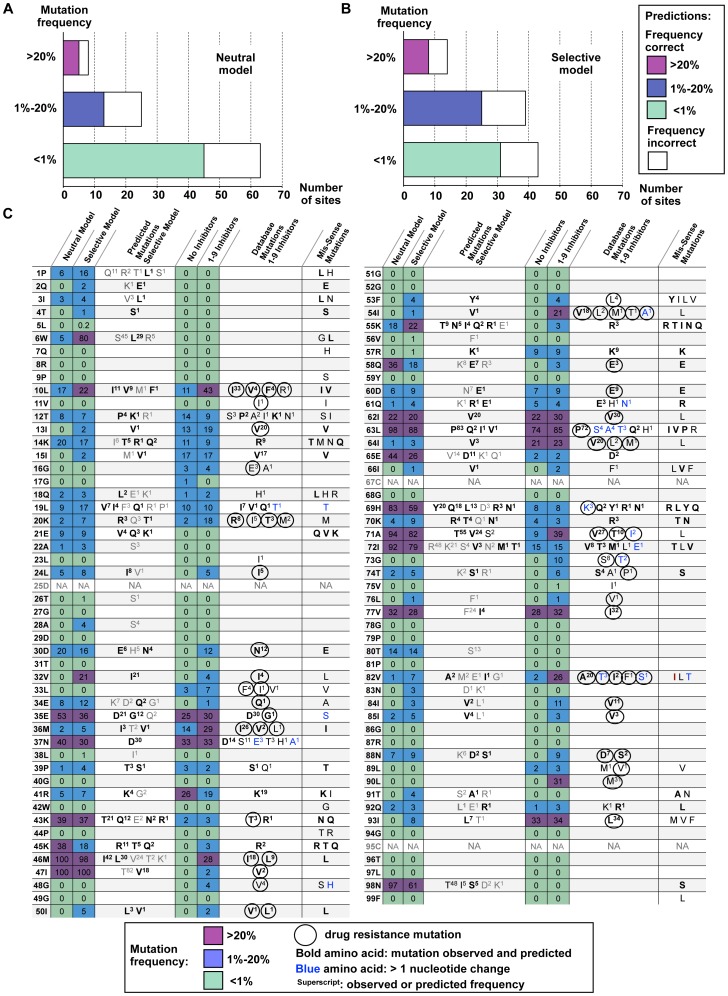
Predicted and observed HIV-1 protease mutational tolerances. (**A**) Bar plots representing the total number of protease sites within the Stanford database observed to mutate at low (<1%, lower bar), moderate (1%–20%, middle bar) or high (>20%, upper bar) frequencies before treatment with protease inhibitors. The number of protease sites predicted to have mutational frequencies that match the database frequencies for the neutral model are colored in light green (lower bar), blue (middle bar) and magenta (upper bar). Sites that were mis-predicted are colored in white. (**B**) As in (A), but for the selective model. (**C**) Overall mutational tolerance and individual mutation frequencies for the analyzed 96 HIV-1 protease sequence positions. Column 1: Sequence position and residue type present in the consensus sequence. Columns 2 and 3: Overall mutational tolerance predicted by the neutral and selective models, respectively, expressed as the % non-native residue types observed at each position. Color code as in (A). Column 4: Individual residue types predicted to be tolerated by the selective model. Columns 5 and 6: Overall mutational tolerance observed in the Stanford HIV database in the absence and presence of inhibitors, respectively, expressed as the % non-native residue types observed at each position. Color code as in (A). Column 7: Individual residue types observed to be tolerated under inhibitor treatment. Column 8: Functional mutations obtained from mis-sense mutagenesis [16]. Residue types that are predicted by the selective model and also observed (columns 7 and 8) are shown in bold typeface. Residue types that are greater than one nucleotide mutation away from the native residue type (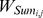 = 0), are noted in blue. Superscripts in columns 4 and 7 show predicted and observed frequencies (not available from the mis-sense mutagenesis experiment) rounded to the nearest 1%. “NA" indicates protease sites for which mutational tolerance was not predicted. Protease drug resistance mutations (DRMs) are denoted by circles (note that DRMs with frequencies lower than .5% are not shown; therefore, only 62 of the 71 documented DRMs [23] are shown).

Approximately 2/3 of protease sites (63 out of 96) within the Stanford HIV-1 Database sequences [15] appeared largely intolerant to mutation prior to inhibitor treatment (**Figure 2A**; intolerance to mutation defined as a mutation frequency of <1%). Further, about half of protease sites within the database (43 out of 96) were largely intolerant to mutations under inhibitor treatment (**Figure 2B**). The neutral and selective models correctly identified the majority of these intolerant protease sites (**Figure 2A–B**; 45/63 and 31/43, respectively).

Within the database sequences, only a few protease sites displayed high mutational tolerance (**Figure 2A–B**; 8 and 14 sites, in the absence and presence of inhibitors, respectively; high mutational tolerance defined as a mutation frequency >20%). The neutral and selective models correctly identified over half of these frequently mutated protease sites (**Figure 2A–B**; 5/8 and 8/14 sites, respectively), including five sites that displayed high mutational tolerance in both a neutral setting and under selective pressure (**Figure 2C**; 35E, 37N, 62I, 63L, and 77V). Importantly, the individual mutations observed in the Stanford database were also correctly predicted for many of the frequently mutated sites (**Figure 2C**; bold residues in 4^th^ and 7^th^ columns). Similar results were observed at sites within the database with moderate mutational tolerance; these sites were often correctly predicted by both the neutral and selective models (**Figure 2C**; 12T, 14K, 18Q, 19L, 20K, 39P, 60D, 61Q, 70K, and 92Q; moderate mutational tolerance is defined as amino-acid variation between 1–20%). Therefore we conclude that the models can, in many cases, recapitulate both protease sites and individual protease mutations that are functionally tolerated (the results for the neutral model are shown in **Figure S1**).

**Figure 3:**
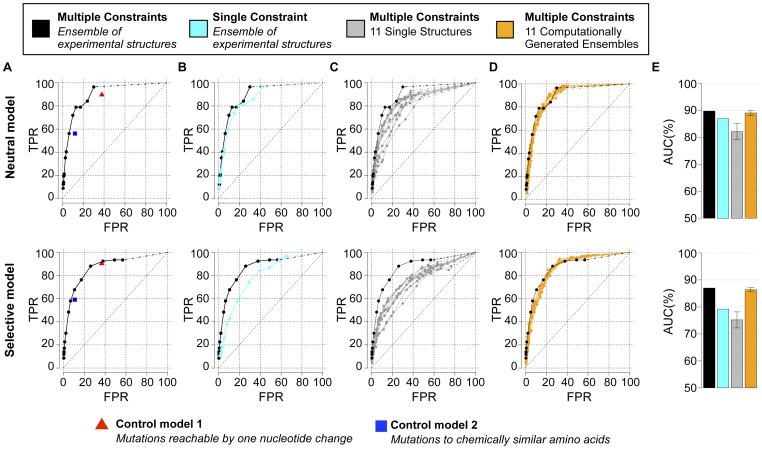
Model performance for protease and importance of specific model features: multiple constraints and backbone flexibility. (**A**) ROC curves for predictions using three functional constraints and a crystallographic ensemble of protease structures are shown for the neutral (top) and selective (bottom) models. For reference, these curves are also duplicated in panels B–D. (**B**) ROC curves for predictions using fold stability as a single constraint and a crystallographic ensemble of protease structures are shown for the neutral (cyan, top) and selective (cyan, bottom) models. (**C**) ROC curves for predictions using three constraints and a single crystallographic protease structure are shown for the neutral (grey, top) and selective (grey, bottom) models. Curves are shown for 11 single protease structures. (**D**) ROC curves for predictions using three constraints and a computationally generated ensemble of protease structures are shown for the neutral (orange, top) and selective (orange, bottom) models. Curves are shown for 11 computational ensembles each generated from one of the 11 single protease structures used in (C). (**E**) AUC values are shown for each of the ROC curves depicted in (A–D). For ROC curves in (A–D), true positive tolerated mutations are defined as those observed with a frequency above 1% in the Stanford database (57 and 93 mutations for the neutral and selective models, respectively). The subset of all amino acids reachable by one nucleotide change and the subset of all amino acids that are chemically similar to the native are denoted by a red triangle and blue square, respectively (see text). Dashed lines connect the last ROC value (lowest frequency threshold) and the (100%, 100%) point.

To quantify the overall ability of the neutral and selective models to recapitulate individual mutations observed in the Stanford database, we used two standard metrics: (1) We computed a Receiver Operating Characteristic (ROC) curve by calculating the true positive rate (TPR) and false positive rate (FPR) of identifying protease mutations observed within the Stanford database above a threshold frequency of 1% and (2) we calculated an Area Under the Curve (AUC) for each ROC plot (**Figure 3**). Both the neutral and selective models recapitulated many HIV-1 protease database mutations without incorrectly predicting a large number of false positives (**Figure 3A and 3E**; black curve and black bar). Commonly, a model with no predictive power will have a ROC curve that is a diagonal line and an AUC value of 50%. We chose two naïve mutation tolerance prediction models as additional references. In control model 1, each site can tolerate all mutations that are accessible by a single nucleotide change from the consensus sequence. In control model 2, each site can tolerate amino acid types chemically similar to the native amino acid (see Methods). Control model 1 predicted the majority of the experimentally observed mutations (TPR ∼90%, red triangle in **Figure 3A**). However, a large number of non-observed mutations were incorrectly predicted as tolerated (∼37% FPR). The computational models had a lower FPR at the same TPR. Control model 2 rarely predicted tolerance to mutations that were not observed within the database (∼11% FPR), but did not capture tolerance to many database mutations (∼60% TPR, blue square in **Figure 3A**). The computational models ranked more mutations correctly at the same FPRs.

In addition to recapitulating database mutations found in either neutral or selective conditions, our prediction scheme was also successful in recovering literature-documented drug resistance mutations (DRMs) for protease. The comparison between predictions of the neutral and selective models (**Figure 2C**) yielded 18 sites that showed increase in mutation frequency (rare/moderate to moderate/high), 9 out of which contain previously characterized DRMs (as listed in [23], see circles in **Figure 2C**). Thus comparing predictions from the neutral and selective models may, in some cases, allow for identification of sites that contain drug resistance mutations.

### Model over-predictions and under-predictions

Overall, the agreement between the individual mutations appearing within the database and the mutations predicted as tolerated by the models was strong (**Figures 2C**, **3A** and **Figure S1**). Nevertheless, several notable under- and over-predictions were observed.

Under-predictions of mutational tolerance by the neutral model were most notable at 10 sites (**Figure 2C**, 2^nd^ and 5^th^ columns; 13I, 15I, 16G, 33L, 36M, 41R, 57R, 64I, 89L, and 93I). The same 10 sites were also under-predicted for the selective model, with additional under-predictions occurring at 8 sites (**Figure 2C**, 3^rd^ and 6^th^ columns; 10L, 20K, 48G, 54I, 73G, 82V, 84I, and 90L). At most of these sites, the specific mutations observed in the Stanford database were correctly identified, but the predicted frequencies of mutation were significantly less than experimentally observed (**Figure 2C**; 4^th^ and 7^th^ columns). Notably, almost all under-predicted sites contained DRMs (see circles in **Figure 2C**; exceptions are 15I, 41R and 57R). Under-predictions may result from errors in the Rosetta energy model or from the inability to correctly capture structural changes in response to sequence changes.

Over-predictions of mutational tolerance occurred primarily within the beta-sheet pairing of the dimer interface (1P, 3I, 6W, 98N), three sites in the dimer flaps (45K, 46M and 47I), and several surface sites (21E, 35E, 43K, 55K, 58Q, 65E, 69H, and 72I, **Figure 2C**). DRMs were relatively rare within sites that were over-predicted, although they did occur at two sites within the protease flaps (46M, 47I) and at surface sites (35E, 43K, 58Q, and 69H; circles in **Figure 2C**). As with under-predictions, model over-predictions could be due either to inaccuracies of the Rosetta model or additional functional pressures not captured. The high predicted frequency of mutation at sites 46 and 47 likely occurred due to the presence of a clash with one of the modeled substrate peptides at these sites. Thus, predictions at these two sites might be improved if a crystallographic structure of protease bound to this modeled peptide was available. In addition, Rosetta often performed poorly at predicting mutation frequencies at polar exposed sites. This poorer performance highlights known difficulties in accurately modeling the energetics of polar interactions. Furthermore, despite the inclusion of two terms to disfavor mutations away from polar residues, we may not correctly capture other pressures acting particularly on surface residues, such as selection against aggregation.

As described above, we noted several instances where the selective model predictions did not agree with the mutations observed in the HIV-1 protease database sequences. However, we found that some predictions instead agreed with mutations shown to be tolerated in an experimental study of single mis-sense mutations [16] (**Figure 2C**, bold residues in 8^th^ column). This finding suggests that the selective model might capture protease mutational tolerance not yet observed at high frequency within the database sequences. In support of this idea, we note that three mutations recently identified in the presence of inhibitors (M46V, F53Y, and N83D) [24], [25] were predicted as tolerated by the selective computational model (**Figure 2C**, 4^th^ column). All three newly identified mutations were not yet found within the protease database sequences at appreciable frequencies.

### Energetic analysis of known drug resistance mutations (DRMs) in HIV-1 protease

As described above, differences observed between the selective and neutral models can be used to recapitulate and predict DRMs. In this section we examine in detail the ability of the model to recapitulate tolerance for 71 previously characterized DRMs. We used a list of mutations from [23] and their grouping into major and minor DRMs. Both groups show an increased frequency of mutation after inhibitor treatment, but only major DRMs have been directly implicated in causing resistance to inhibitors.

**Figure 4:**
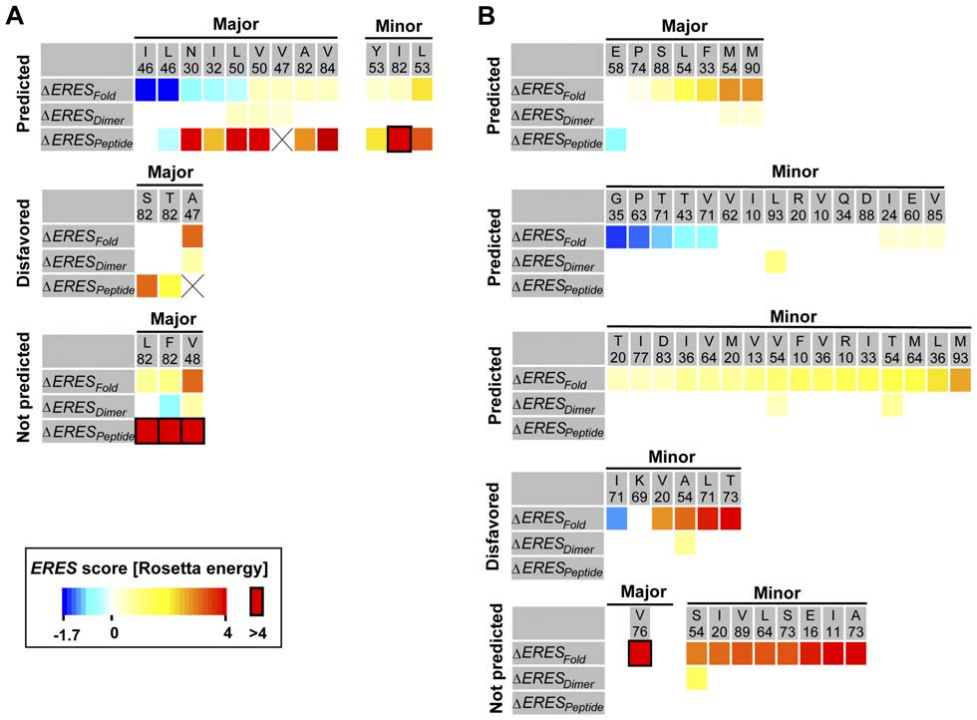
Predicted energetic contributions of HIV-1 protease DRMs. (**A**) DRMs within 4 Å of the substrate-binding site [23]. Predicted changes in *ERES_Fold_*, *ERES_Dimer_*, and *ERES_Peptide_* scores are relative to the *ERES* scores of the native residue type. *ERES_Peptide_* scores are represented by the change in the sum of *ERES* scores for all 10 peptides before and after introducing the mutation. *ERES* scores are given in color codes, from −1.7 to 4 (blue to red), and >4 (framed red boxes) in Rosetta energy units (approximating kcal/mol), and columns are sorted in ascending order of the *ERES_Fold_* scores. Mutations denoted as “Predicted" and “Not Predicted" were predicted by the selective model to have >0.01% and ≤0.01% frequencies, respectively. Mutations that required more than one nucleotide substitution are denoted as “disfavored". Boxes with “X" indicate clashes in the wild-type structure. (**B**) As (A), but showing DRMs outside of the substrate-binding site. Major and minor DRMs are as defined in the text.

The selective model permits mutations near the protease inhibitor binding-site by weakening constraints on the protease dimer and substrate-binding interface. We first analyzed whether the selective model predicts tolerance to DRMs located within the inhibitor-binding site. Of the 18 DRMs near the substrate-binding site, 12 were predicted as tolerated by the selective model (**Figure 4A**; 3 DRMs were disfavored by the model as they required more than a single nucleotide change from the consensus sequence). Not surprisingly, most DRMs within the substrate-binding site were predicted to have mild-to-moderate destabilizing effects on binding of at least one of the 10 endogenous peptide substrates (**Figure 4A**, red coloring). The three DRMs not identified by the selective model were predicted to highly destabilize binding of at least one peptide (**Figure 4A**, red boxes; 82L/F, 48V). In contrast, effects on fold and dimer stability of the DRMs within the inhibitor-binding site were predicted as mostly energetically favorable or neutral (**Figure 4A**, blue and beige coloring; 47A, 48V and 53L are notable exceptions). At least one mechanism to compensate for substrate binding destabilization is known. Peptide sequences cleaved by HIV protease can co-evolve with the appearance of DRMs such that mutations within the cleavage sequences counteract the predicted losses in substrate binding affinity [26], [27], [28]. Although the selective model does not directly mimic this mechanism of co-evolution, it correctly predicted tolerance to most documented DRMs within the protease inhibitor-binding site.

We next examined DRMs known to occur outside of the protease substrate-binding site. Here, the selective model correctly predicted mutational tolerance towards almost all major DRMs and towards the majority of minor DRMs (**Figure 4B**; 7/8 and 31/45, respectively; note 6 minor DRMs were disfavored by the model). In the cases where the model did not predict a DRM to be tolerated, it was because the mutation was calculated to strongly destabilize the protease fold (**Figure 4B**, red coloring). These predicted destabilizing effects of some mutations may need to be compensated for by other co-occurring mutations. Consistent with this hypothesis, 4 out of the 12 predicted destabilizing DRMs (close and far from the substrate binding site) occurred in the 53 most statistically significant correlated pairs of mutations observed after protease inhibitor treatment [29]. Even though the selective model currently cannot account for correlated mutations, it nevertheless correctly predicts tolerance towards a considerable number of DRMs outside of the protease-binding site.

We next examined the contribution of stabilizing mutations to DRMs in HIV protease. This analysis was based on a set of 62 out of the 71 documented DRMs, which had a frequency of >0.5% in the Stanford HIV database. In total, 11 of the DRMs were predicted to stabilize the protease fold, both within (30N, 32I, 46I/L, 50L **Figure 4A**) and outside (35G, 43T, 63P and 71V/I/T, **Figure 4B**) the binding site. Interestingly, DRMs at sites 30, 32 and 50 are predicted to have a favorable effect on fold stability, and a destabilizing effect on peptide binding. We asked whether DRMs that are predicted to have a fold-stabilizing effect (out of all 62 DRMs that are both documented and predicted) are over-represented relative to any documented protease mutation predicted to have a fold-stabilizing effect (out of all possible protease mutations reachable by a single nucleotide change from the consensus sequence). We found that there is a significant overrepresentation of DRMs that are predicted to be stabilizing (Δ*ERES_Fold_*<0): 17.7% (11/62), in contrast to only 10% (72/705) of all protease mutations observed in the HIV-1 database reachable by a single nucleotide change (*p* value = 1.43E-7, Mann-Whitney test). One possible reason for the overrepresentation of stabilizing DRMs is that these sites reside in special locations (such as buried sites that generally contribute more to stability) in the protein structure. We thus calculated the percentage of buried and exposed DRMs and compared these values to the percentage of buried and exposed residues of all documented protease mutations (**Figure S2**). We found no significant difference in the burial of positions at which DRMs appear. In addition, we studied a list of 33 frequent DRMs that often occur in combination (extracted from the Stanford HIV database, **Table S5**). Assigning our calculated *ERES_Fold_* scores for these mutations, we found that 22/33 of the co-occurring mutations included a combination of at least one destabilizing and one stabilizing mutation. These analyses suggest that the modeled stabilizing DRMs may play a role in drug resistance by compensating for the destabilizing effects of other mutations.

### Importance of specific model features: multiple constraints and backbone flexibility

We next analyzed whether two key features of the model – incorporating multiple constraints and using backbone ensembles – contributed to prediction performance, using the ROC and AUC metrics introduced above. We first asked whether the model we present, which incorporates fold, dimer and peptide constraints for HIV-1 protease, would outperform a simpler model that considers only fold stability. To do so, we recalculated mutational tolerance at every protease site, but this time we used only the 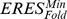 scores for each point mutation and we set all the 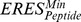 and 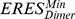 terms to zero (“single constraint model"). The predictions of mutational tolerance from this single constraint model were less accurate than the original multiple constraint model, at least under selective conditions (**Figure 3B and 3E**; cyan curves and bars). Thus, incorporating multiple constraints may be particularly useful for modeling selective pressure, because it allows weakening of certain constraints (such as dimer stability and substrate binding) over others.

We next tested how accurately protease mutational tolerance would be predicted if only a single protease structure was used. To do so, we tested a “single structure model", in which we made 263 independent calculations of HIV-1 protease mutational tolerance. In each set of predictions, we used the *ERES_Fold_* and *ERES_Dimer_* scores calculated from a single backbone structure rather than finding the minimum 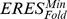 and 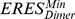 scores calculated over the entire ensemble of structures (identical 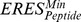 scores were used in all cases, see Methods). When we compared ROC curves and AUC values obtained from predictions made using single protease structures (**Figure 3C** grey curves shown for 11 structures; **Figure 3E** grey bars) to model predictions made using the ensemble of crystal structures (black curves and black bars), we again observed consistently poorer model performance. This suggests incorporating backbone variability by making predictions over an ensemble of backbone structures can be important for correctly predicting protease mutational tolerance.

### Model performance using computationally generated conformational ensembles

HIV protease has been particularly well characterized and hundreds of solved crystal structures exist within the Protein Data Bank. Many of these protease crystal structures originally contained point mutations. Thus the improvement seen in predicting mutational tolerance using the ensemble of protease crystal structures could have been influenced by the original presence of these point mutations (while all mutations were computationally reverted to the consensus sequence at the start of our simulations, any backbone structural changes present in the mutated structure remained, see Methods). Furthermore, other proteins may not have comparably large ensembles of experimental structures and thus the method we describe here could, for this reason, be less applicable.

To address both these issues, we next tested whether accurate predictions of mutational tolerance could be made using a computationally generated, rather than an experimentally determined, ensemble of protease backbones. To ensure that the computational ensemble did not contain “structural memory" of point mutations present in the original crystallographic ensemble, we selected as templates 11 protease crystal structures that did not contain mutations from the consensus sequence. From each of the 11 templates, we used a computational method termed “backrub" to generate an ensemble of 400 protease structures [20], [30] with “near-native" backbone conformations (ensemble members had Cα RMSDs of 0.2 to 0.6 Å to the original starting template structure). We then repeated the calculations of mutational tolerance using each computational ensemble as described for the ensemble of experimentally determined structures.

Remarkably, the same crystal structures that had resulted in poorer ROC curves and AUC values when considered as single structures (11 grey curves and grey bar, **Figure 3C and 3E**) now showed improved results when the structures were used as starting templates for a computationally generated ensemble (11 orange curves and orange bar, **Figure 3D and 3E**). Furthermore, ROC curves and AUC values for predictions made using computationally generated ensembles were almost identical to those originally made using the ensemble of experimentally determined crystal structures (compare black and orange curves and bars, **Figure 3D and 3E**). Therefore, while increasing the computational cost linearly with the number of backbones (see **Text S1** for estimates on computational time), backbone ensemble calculations can result in considerably better prediction than when using only a single backbone.

### Structural and energetic analysis for representative mutations

**Figure 5:**
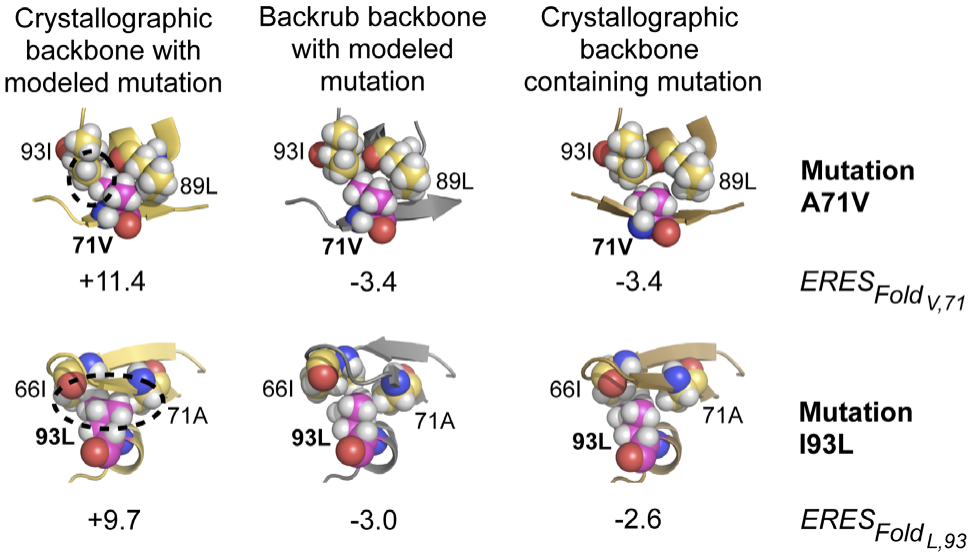
Structural and energetic effects of modeling backbone flexibility. Shown are space fill representations of the environment of two mutations: A71V (top) and I93L (bottom); yellow: carbon; magenta: carbon of mutated residue; white: hydrogen; red: oxygen; blue: nitrogen. Left: Mutations A71V and I93L modeled onto a crystallographic structure that did not contain a mutation (PDB code 1VIK) result in large to moderate steric clashes (unfavorable *ERES* scores). Middle: The same mutations modeled onto structures that had been computationally generated using the backrub protocol; the steric clashes are relieved (negative *ERES* scores). Right: *ERES* scores and structures for experimentally determined structures that contained the A71V or I93L mutations (2FDD and 2R5P, respectively) were close to the modeled *ERES* scores and structures (middle). *ERES* scores for each structure represent only the *ERES_Fold_* contribution from the single chain depicted.

To gain insight into how structural flexibility might have resulted in improved predictions of mutational tolerance, we examined the model predictions in more detail. **Figure 5** shows two mutations as examples where backbone flexibility appeared to be crucial for correctly predicting tolerance to mutations observed in the Stanford database. When mutations A71V and I93L were individually modeled onto HIV-1 protease fixed backbone structures crystallized in the absence of any mutation, large to moderate clashes resulted (**Figure 5**, left). In each case, the clashes could be resolved when the same mutation was modeled onto a backbone computationally generated from an unmutated starting structure using the backrub method (**Figure 5**, middle). The mutations modeled onto the computationally generated backbones had structures and *ERES_Fold_* scores close to those seen in experimentally determined structures that had originally contained the mutation (**Figure 5**, right). These results suggest that backrub ensembles, even though they were generated in the absence of mutations, can capture sufficient protein conformational variability to accommodate amino acid changes [20], [30].

**Figure S3** confirms that the mutations 71V and 93L were predicted as tolerated when modeled onto either experimental or backrub ensembles, but never when modeled onto a single fixed backbone of the consensus sequence (similar behavior was also observed for mutations 24I and 77I, **Figure S3**). We note that a few mutations within the Stanford database sequences that had been poorly predicted when using the ensemble of experimentally determined structures were found to be tolerated when using computationally generated ensembles (*e.g.* 33F and 12S, see **Figure S3**).

### Extending the model to test mutational tolerance in a second protein: HIV-1 reverse transcriptase

To test the applicability of our model, we chose another protein system, the HIV-1 reverse transcriptase. This RNA-dependent DNA polymerase transcribes the single-stranded retroviral RNA genome into a double-stranded proviral DNA. Reverse transcriptase is a heterodimer built of the p66 subunit (560 residues) and the p51 subunit, which has an identical sequence to the first 440 residues of p66. The unique C-terminal part of the p66 subunit comprises an RNaseH domain. Similar to the protease system, many reverse transcriptase structures have been determined, many pre/post drug treatment mutations are catalogued in the Stanford database [15] and mutational tolerance prediction can be made using both fold and dimer stability as functional constraints. Nonetheless, the reverse transcriptase model has several limitations. There are fewer crystal structures than for protease (see **Table S1**) and there are stretches of sequence with missing density in these structures. The substrates of reverse transcriptase are DNA/RNA hybrid molecules, for which interaction energy calculations are less established than for protein-protein interactions. We therefore did not consider reverse transcriptase residues in the interface with nucleic acids. In addition, model predictions could not be verified for the RNaseH domain, since mutational data are too sparse in this protein segment (see Methods). In sum, we evaluated our analysis based on an ensemble of 91 structures and 656 of the 1,000 residues in the reverse transcriptase heterodimer (still a much larger number of residues than in HIV protease; note that while some residues are excluded from the analysis, all protein residues present in the structures were used in the calculations). We repeated all mutational tolerance calculations as described for protease, calculating *ERES_Fold_* and *ERES_Dimer_* scores for every structure within the ensemble (*ERES_Peptide_* could not be computed for reverse transcriptase that does not have peptide substrates). Otherwise the model parameters determined for protease were used unchanged for reverse transcriptase. Mutations were made simultaneously for every shared sequence position in the p51 and p66 subunits, while the p66-specific RNAseH domain sites were mutated only on the p66 subunit. The detailed results for the modeled versus observed mutational tolerance for reverse transcriptase are given in **Figure S4** (neutral model) and **Figure S5** (selective model).

**Figure 6:**
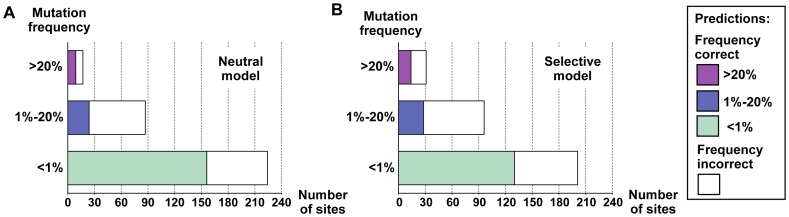
Recapitulation of reverse transcriptase mutational tolerance by the neutral and selective models. Panels **A** and **B** have same representation as in Figures 2A and B.

As was observed for protease, a sizeable number of reverse transcriptase sites have low mutational tolerance, and a rather small number of sites were frequently mutated (224 and 17 sites, see **Figure 6A**). The neutral model correctly identified the majority of these sites (70% and 53% for the rarely mutated and frequently mutated sites, respectively). In contrast, the performance of the selective model for reverse transcriptase was poorer: 130/201 sites that rarely mutate were correctly predicted, and 14/31 sites that frequently mutate (see **Figure 6B**). Under-predictions were seen at five out of 17 sites (177D, 211R, 329I, 334Q and 376A), while over-predictions were seen for 42 out of 224 (19%) sites. Thirteen out of these 42 sites are exposed polar residues (as for protease, Rosetta performed poorly at predicting at polar exposed sites).

Many sites with over-predicted mutational tolerance are in protein segments that rarely mutate due to constraints likely not captured in our prediction scheme. For example, 17 of the over-predicted sites are located in the Palm domain (positions 86–119 and 151–244). Within it, sites 88W, 111V, 113D, 116F, 182Q and 233E were shown to be involved in primer loading [31]. Another over-predicted stretch of residues spans positions 216 to 243 (the “primer grip") that is involved in positioning the primer's terminus [32]. This region is almost invariant in the neutral data and is known to mutate after drug treatment (as shown in the selective settings – both in the database and the modeled data). An additional over-predicted segment spans position 251 to 271 (the ‘helix clamp’) that is conserved among other nucleic acid polymerases [33]. Several residues within these regions were not directly in contact with nucleic acid in any of the available structures but were previously shown to be important for the catalytic cycle of reverse transcription, providing a possible explanation for the over-predictions.

**Figure 7:**
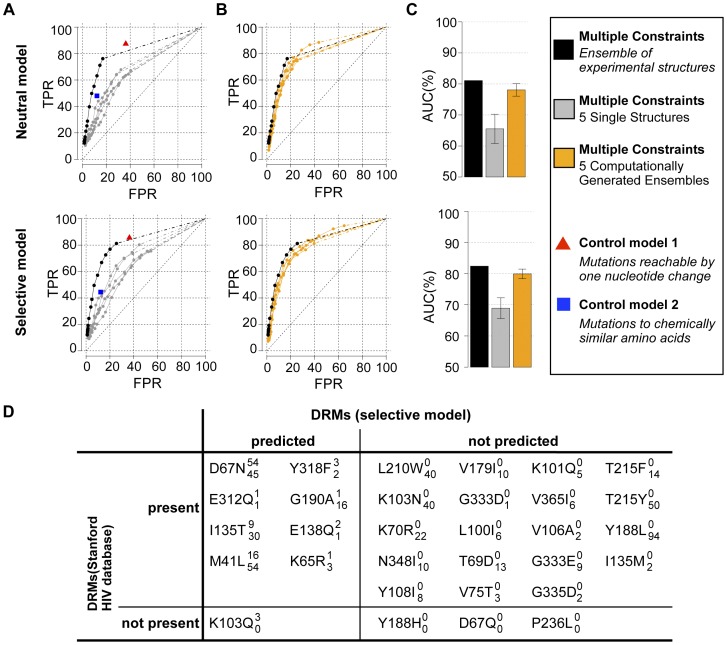
Performance of specific model features and DRMs for reverse transcriptase. Data representation in panels **A**, **B** and **C** is the same as in Figure 3C, D and E. (**D**) Recapitulation of reverse transcriptase DRMs by the HIV database and the selective model: A set of 31 literature-documented DRMs of reverse transcriptase [63], [64], [65], [66], [67] was categorized according to whether or not they are present in the Stanford HIV database (post-drug treatment data) and whether or not they are recapitulated by the selective model. Subscript and superscript numbers list mutation frequencies according to the HIV database and the selective model, respectively.

As with HIV-1 protease, we calculated ROC curves and AUC values for the reverse transcriptase model predictions to quantify overall performance (**Figure 7**). The ROC curves show that the computational model correctly ranked many mutations tolerated by HIV-1 reverse transcriptase. AUC values are generally slightly lower for reverse transcriptase than for protease, but exceed 80% (black bars, **Figure 7C**). In accordance with the results obtained for protease, predictions of mutational tolerance made using any single reverse transcriptase structure were worse than using an ensemble of experimentally determined structures or backrub ensembles computationally generated from a single template structure (grey, black and orange curves and bars in **Figure 7A–C**). In conclusion, although mutational tolerance predictions for reverse transcriptase were less accurate than for protease, the results still demonstrate reasonable agreement with mutations observed in the database. The application of the model to reverse transcriptase also confirms the notion that using either ensembles of experimentally determined or computationally generated structures improve predictions over using single structures.

Finally, we examined the ability of the selective model to predict DRMs for reverse transcriptase. Nine of the 31 published DRMs were recapitulated by the model. Interestingly, the K103Q DRM, which was not present in the Stanford database (at the time of the database download), was correctly predicted by the model (see **Figure 7D**). The overall performance of DRM prediction by the selective model was weaker than for protease. Several factors may account for this discrepancy: The number of documented DRMs is lower for reverse transcriptase, and DRM positions are not as evenly distributed over the protein structure as in protease (**Figure S6**). Hence, down-weighting constraints overall leads to many over-predictions in reverse transcriptase. Moreover, the weights of the model were parameterized using the HIV-1 protease data. In addition, the NNRTI inhibitor binding site is not as close to the dimer interface (where constraints are weakened in the selective model) as in protease. Nonetheless, as for protease, some predictions by the selective model might represent resistance mutations yet to be discovered.

## Discussion

We have shown that an all-atom, computational model that incorporates structural and functional constraints on mutational tolerance is able to predict a considerable fraction of the observed tolerated sequence space of HIV-1 protease and reverse transcriptase. The model uses a previously published and established energy function that, importantly, was complemented with incorporation of protein backbone flexibility. The Rosetta energy function has been shown previously [34] to perform comparably to other methods [35], [36], [37] in predicting changes in protein stability upon point mutations. The model parameters in **Equation 1** were optimized using data on HIV-protease by adjusting the relative frequencies with which the various structural and functional constraints operated (as well as a solubility parameter, see Methods). However, there was no explicit parameterization with respect to the actual identities of amino acids selected as tolerated at each protease site, which we use as an evaluation metric in the ROC analysis. Furthermore, after the model parameters were optimized for protease, identical parameters were applied to the much larger protein reverse transcriptase, albeit with a moderate reduction in performance.

The comparison of the predictions of the model with the database mutations, as well as the comparison between the two model systems, reveals both strengths and weaknesses of our approach. Considering multiple structures improved predictions for both proteins. However, developing parameters for one system, such as protease, may make the predictions less applicable to other proteins. This is especially obvious for the dimer and peptide binding constraints used here. These constraints work well for modeling selective pressure in the presence of inhibitors for protease (where inhibitors bind in the peptide binding site, located in the dimer interface), but not for reverse transcriptase. Moreover, the model is likely to fail for regions where important constraints were not included, such as interaction interfaces of reverse transcriptase with nucleic acids. Similarly, HIV protease and reverse transcriptase may bind other partners, as indicated by recent large-scale mapping of interactions of HIV proteins with factors in the human host [38]. If these interactions are not adequately modeled, the sequence constraints they impose may not be correctly captured. Nevertheless, there is overall encouraging agreement between observed and predicted mutational tolerance, suggesting that models similar to the one we developed could be applicable to other proteins.

Errors in the Rosetta energy function are likely responsible for both model over- and under-predictions. This behavior is particularly apparent for mutations to and from polar residues, due to the difficulty of modeling the balance of electrostatic interactions and solvation. Furthermore, the model considers only single, independent mutations, whereas sites that were under-predicted may require the presence of additional compensatory mutations. Correlated mutations occur with both HIV protease and reverse transcriptase drug resistance mutations [39]. For example, the protease mutations 30N and 88D are known to co-vary and while the model predicts mutational tolerance towards both of these mutations, the frequencies predicted are less than seen in the database sequences for each mutation. In such cases, modeling the effects of double mutations may improve predictions. While such a double-mutant analysis is challenging, repeating the selective model calculations using a finite set of double mutations (see **Text S1**) resulted in predicted increases of individual mutation frequencies at 10 of the previously under-predicted protease sites (10L, 20K, 33L, 36M, 41R, 57R, 64I, 82V, 84I, and 93I; **Figure S7**).

A strength of our approach is the improved prediction accuracy when using backbone ensembles. This is observed for both protein model systems. Our results underline that incorporating this backbone variability is important for predicting mutational tolerance, particularly when using all-atom force fields that model atomic packing interactions sensitive to precise details and small steric clashes. We show that predictions made from a computationally generated ensemble can be just as accurate as predictions using an ensemble of experimentally determined structures. This finding is notable, as the conformational variation within the ensemble of experimentally determined backbones included changes induced by substrate and inhibitor binding, as well as structural changes in response to single and multiple mutations. In contrast, the computational ensembles were generated from single structures with few or no mutations, to avoid such “structural memory".

The use of crystallographic ensembles to model protein conformational flexibility has been described and shown to be consistent with molecular dynamics simulations [21], elastic network models [40] and protein dynamics detected using nuclear magnetic resonance [41]. In this work, structural variation calculated over the computationally generated backrub ensembles is similar to variation calculated over the ensemble of experimentally determined structures (**Figure S8**). Previous studies on a variety of other protein systems have found that computationally generated backrub ensembles improve predictions of protein dynamics [19], [42], conformations of single point mutations [20] and sequence diversity in proteins and protein-protein interfaces [18], [22]. Taken together with these results, it is plausible that backrub ensembles sample a significant portion of conformations accessible to proteins.

While the magnitude of stabilizing and destabilizing energetic trade-offs predicted by the model for each individual mutation is only an estimate, the patterns of compensatory effects and functional tradeoffs may nevertheless be informative. There is widespread evidence for the general trend of mutations that confer new functions to destabilize a protein [43], [44]. In contrast, our analysis shows that stabilizing mutations are in fact overrepresented in DRMs relative to all possible mutations in protease that are reachable by a single nucleotide change. Therefore, DRMs arising in viral populations may not conform to the classical definition of ‘new function’ mutations (*e.g.* in [45]). Instead, a significant fraction of DRMs may belong to the subset of mutations described in [45] that increase protein stability. This is consistent with the hypothesis that several DRMs function to compensate for other co-existing destabilizing mutations that directly affect inhibitor binding.

For HIV-1 protease, the selective model suggests hypotheses about the effects of specific mutations on the stability of the protease fold, dimer interface and substrate binding that, in some cases, can be confirmed using existing experimental data. For example, the model predicted large substrate destabilization effects for I47A and V82A/F/T. These mutations are known to display increased replication in viruses with mutations in either the Nucleocapsid/p1 or p1/p6 cleavage sites [28], [46], [47]. This finding suggests that cleavage site mutations may compensate destabilizing effects at the substrate-binding interface predicted by the model. In another example, incorporating A71V (predicted by the model to stabilize the protease fold) into double and triple mutants with reduced replicative ability (containing either 36I/54V or 36I/54V/82T, all predicted to be destabilizing) has been shown to improve replication to better than wild-type levels [48].

The approach we present here differs from other studies that have characterized the structural [49], [50], functional [51], [52], [53], [54], [55] and energetic effects [55] of HIV-1 protease mutations on inhibitor binding (and similar studies applied on reverse transcriptase [56], [57]). Instead, we make predictions of the mutations tolerated by HIV-1 protease and reverse transcriptase structure and function without explicitly considering inhibitor binding. A possible limitation of this approach is that mutations at sites that directly interact with a protease inhibitor may be under-predicted even in the selective model, since no benefit is given to mutations that specifically destabilize protein-inhibitor interactions. On the other hand, our model should have the advantage of predicting protease mutational tolerance prior to knowledge of any specific inhibitor structures. While some drug resistance mutations are shared among inhibitors, new drug resistance mutations have appeared with the introduction of each clinical drug. The model we present here could be useful in the prediction of yet undiscovered resistance mutations by suggesting mutations structurally and functionally tolerated that would be free to contribute towards the destabilization of new inhibitors.

In conclusion, our results, along with the observation that RosettaDesign simulations using flexible backbone ensembles capture sequence diversity in phage display experiments [18], [22], [58] and protein families [19], suggest that the model presented here for protease and reverse transcriptase may be applicable to other proteins. Moreover, while we initially validated our model using experimentally determined structures of HIV-1 protease and reverse transcriptase solved under a variety of experimental conditions, we have also shown that computationally generated structural variability from a single structure can produce comparable model accuracy. Thus, the model we present here could be extended to predict mutational tolerance in other systems (where only a single structure may be available) to yield insights into the relationship of structure, function, and tolerated sequence space. In practical terms, prediction of the nearly neutral space of sequences consistent with a given structure and function may be exploited in the experimental design and construction of proteins with modified and new properties.

## Methods

### Protease and reverse transcriptase consensus sequences

The following HIV-1 protease and reverse transcriptase sequences for subtype B, defined by the Stanford HIV database as the consensus sequence, were used throughout this work. Sequence positions in lower case were excluded from model predictions (see below).

Protease:

PQITLWQRPLVTIKIGGQLKEALLdTGADDTVLEEMNLPGRWKPKMIGGIGGFIKVRYDQILIEIcGHKAIGTVLVGPTPVNIIGRNLLTQIGcTLNF

Reverse transcriptase: pispIETVPVKLKPGMDGPKVkQwpltEEkIKALVEIcTEMEKEGKISKIGPENPYNTPVfAiKKkDSTKWRKlVdFrELnKRTQDFWevqlGiPHPAGLKKKKSVTVLdVGDAyFSVPLDKDFRKYTAFTIPSINNETPGIRYQYNVLPqGWkGSpAIFQSSMTKILEPFRKQNPDIVIYQymddLYVGSDLEIGQHRTKIEELRQHLLRWGFTtpdkkhqkeppflwmGYELHPDKWTVQPIVLPEKDSWTVnDIqkLVGkLnwASQIYAGIKvkQLckLLrGtkAlTEVIPLTEEAELELAENREILKEPVHGVYYDPSKDLIAEIQKQGQGQWTYQIYQEPFKNLKTGkyaRMrGahTNDVKQLTEAVQkIATESIVIWGKTPKFkLPIQkeTWEawwteywqatwipewefvntpplvklwyqlekepivgaetfyvdgaanretklgkagyvtdrgrqkvvsltdttnqktelqaihlalqdsglevnivtdsqyalgiiqaqpdkseselvsqiieqlikkekvylawvpahkgiggneqvdklvsagirkvl

In total, we include 96 out of 99 protease sites, and 328 out of 560 reverse transcriptase sites (note that our algorithm was applied on two chains of each of these proteins, meaning 192 amino acids of protease and 656 amino acids for reverse transcriptase). Not included in the analysis were known catalytic site residues in both proteins (1 in protease and 3 in reverse transcriptase [31]). We also excluded all mutations to and from cysteine as modeling the effect of these mutations can be complicated by disulfide bond formation. For protease, this included mutations at the two naturally occurring cysteine sites (67C and 95C) which were relatively rare and never occurred to any other amino acid type with a frequency >1%. Five mutations to cysteine in the presence of inhibitors were also excluded: L63C 2.8%, N37C 1.3%, G73C 0.9%, I84C 0.4% and V82C 0.1%. For reverse transcriptase, we excluded two naturally occurring cysteines (C38 and C280) which were never documented to mutate to any other amino acid type with a frequency >1%. Eleven mutations to cysteine (pre-drug treatment) were also excluded, only one of which occurred frequently: A33C 0.9%, W88C 0.3%, S162C 20.9%, Y181C 0.4%, T215C 0.3%, S251C 0.2%, Q334C 0.4%, G335C 0.3%, F346 0.4%, A376C 0.1% and S379C 1.9%. Additional residues omitted from the reverse transcriptase analysis are 161 residues with insufficient information on mutational tolerance in the Stanford HIV database (**Figure S9**), residues in contact with the RNA substrate (46 positions) and regions of missing densities in the available crystal structures (20 positions).

### Observed mutational frequencies

Frequencies of mutated amino acids in protease and reverse transcriptase observed in patients were obtained online from the Stanford HIV drug resistance database (Genotype-Treatment Correlations/Treatment Profiles, see http://hivdb.stanford.edu/cgi-bin/PRMutSummary.cgi and http://hivdb.stanford.edu/cgi-bin/RTMutSummary.cgi). The settings were as follows - reference profile: subtype B untreated, exclude single occurrences: yes, include mixture: no, one mutation per person. We also compiled a sequence set after inhibitor treatment by using similar settings for protease (# of PIs: 1–9, in addition to the profile settings described above) and reverse transcriptase (#NRTI: 1–7, #NNRTI: 1–4).

### Experimentally determined structures used for predictions of fold and dimer stability

For protease, 262 dimeric crystal structures (see **Table S1**) and one NMR minimized model (PDB code: 1BVG) were used as the ensemble of experimentally determined structures. The majority of structures contained 1–7 mutations (223 out of 263). The crystallographic resolution for structures in the ensemble is within the range of 0.84 to 3.1 Angstroms. For the ensemble of experimentally determined structures of reverse transcriptase, we compiled 91 crystal structures (see **Table S1**) that have a resolution within the range of 1.8 to 3.2 Angstroms and contain 13–24 mutations from the consensus sequence.

### RosettaDesign energy function and sampling

All computational simulations were performed using the Rosetta energy function [17], [59], which is dominated by atomic packing, attractive and repulsive Lennard-Jones interactions, an orientation-dependent hydrogen bonding term [59], and an implicit solvation model [60]. The simulations consisted of sampling and scoring side-chains (taken from a rotamer library that included the native amino acid conformations taken from the starting structures and additional rotamers around the chi1 and chi2 side-chain torsion angles [61]) using a Monte-Carlo simulated annealing optimization protocol (“repacking") as described in [62].

### Preparation of experimentally determined structures

In preparation for calculations of fold and dimer stability, all water molecules, heteroatoms, DNA/RNA nucleotides, bound inhibitors or substrates and hydrogens present in the original PDB structures were removed, and hydrogen atoms were added as previously described [59]. An initial round of minimization of the side-chain torsion angles was performed using the Rosetta energy function, keeping all amino acid identities and backbone coordinates fixed. After this initial minimization, all structures containing mutations from the consensus HIV-1 subtype B sequence (see above) were computationally reverted to the consensus sequence. All side-chains that had at least one atom within 4 Å of any mutated residue were repacked and the structures were side-chain minimized a second time.

In preparation for calculations of peptide substrate binding affinity to HIV-1 protease, a protocol identical to that described above, except leaving all bound substrates present, was used for the 19 crystallographic and model structures listed in **Table S2**. 16 dimeric, crystallographic structures with one of 7 endogenous peptide substrates were used for calculations of substrate binding energy (see **Table S2**). Structural models for each of the three peptide substrate sequences without experimentally determined structures (CTLNF-PISPI, PQITL-WQRPL and VSFNF-PQITL) were generated by computationally threading each peptide sequence onto each of the known 16 crystallographic structures (sequence positions for which there was missing crystallographic density on any of the 16 peptide template structures were omitted) and performing side-chain minimization and repacking as described above. We selected the structural template for which the resulting Rosetta interface energy (the sum of Rosetta energy terms over all pair-wise interactions between residues l and m, where residue l was located on HIV-1 protease and residue m was located on the bound peptide) was the lowest. 1MT9.pdb was found to be the best template for both peptides CTLNF-PISPI and PQITL-WQRPL, while 1F7A.pdb was selected as the optimal template for VSFNF-PQITL. Peptide interface Rosetta scores for the resulting three models (−18.3 to −25) were within the range observed for 16 crystallographic structures obtained with bound peptides (−18.5 to −33).

### Estimation of mutational effects on fold stability, dimer interface stability and peptide binding

Estimates of the effect of mutations on fold stability (*ERES_Fold_*), dimer interface stability (*ERES_Dimer_*) and peptide binding (*ERES_Peptide_*) were calculated using the RosettaDesign energy function and the computational model outlined in Results. The contribution towards fold stability (*ERES_Fold_*) of each mutated residue was estimated by recording the sum of inter- and intra-residue Rosetta energy terms (see **Text S1** for a detailed explanation of the energy terms). The contribution of each mutated residue to stability of the dimer interface (*ERES_Dimer_*) was estimated by calculating only inter-chain pair-wise Rosetta energy function contributions between the mutated residue and neighboring residues on the opposite dimer chain (see **Text S1** for details). The contribution of the mutated residue towards binding interactions with endogenous substrate peptides (*ERES_Peptide_*) was calculated by summing only over pair-wise energy function terms between the mutated residue and the residues of each of 10 bound substrates (as described for the dimer interface).

Each mutation was modeled simultaneously on both chains of HIV-1 protease or reverse transcriptase, and *ERES* scores from both chains were summed. Note that reverse transcriptase is a heterodimer built of the p66 subunit (560 residues) and the p51 subunit, composed of the first 440 residues of p66 (the sequence-identical parts in p66 and p51 adopt different relative orientations of the constituent domains; if each domain is superimposed separately, the average RMSD is 1.18 Angstrom). The unique C-terminal part of the p66 subunit is the RNaseH domain.

For HIV-1 protease, three of the simulations modeling peptide binding required a portion of the HIV-1 protease sequence itself to be a substrate (this occurred for the transframe region and HIV-1 protease cleave site (TF-PR), the HIV-1 protease and reverse-transcriptase cleavage site (PR-RT) and the auto-proteolysis cleavage site (AutoP); see **Table S2**). For these simulations, each relevant mutation was modeled simultaneously onto both chains of the HIV-1 protease scaffold as well as onto the peptide backbone.

Optimal values for the six model parameters (*W_Fold_*, *W_Dimer_*, *W_Peptide_*, *Favor_Native_*, *Favor_Polar_* and *Penalty_Polar→Hydrophobic_*) were selected using a grid search (see **Table S4** for values used) and computing predicted amino acid frequencies over 96 (the catalytic aspartate D25 and cysteine residues C67 and C95 were excluded) HIV protease sites using the minimum *ERES* scores calculated from the ensemble of experimentally determined structures. For each combination of parameters, each of the 96 HIV-1 residue sites was computationally classified as having either low (1–5%), medium (5–20%) or high (>20%) mutational frequency and the number of residue sites correctly matching the experimentally observed mutational frequency bin was calculated. The percentage of sites correctly determined for each bin was then averaged and used to determine a parameter set for both the “neutral" and “selective" computational models. Both the neutral and selective model parameters were applied unchanged to reverse transcriptase.

### Generation of backrub structural ensembles

Computational ensembles of “near-native" backbones were generated starting from one of 11 crystallographic structures of the HIV-1 protease consensus sequence (1A8G, 1EBY, 1HXW, 1IZH, 1PRO, 1SBG, 1VIJ, 1VIK, 4PHV, 5HVP and 9HVP) and one of five structures of reverse transcriptase (1HNI, 1HPZ, 1IKX, 2B6A and 2BAN) by using the previously described backrub protocol [20], [22]. While the protease structures were selected to have the consensus sequence, this was not possible for reverse transcriptase, as all structures contained at least 13 sequence changes from the consensus. For reverse transcriptase, we therefore chose structures that had among the lowest number of mutations (15 to 22) and in addition did not have any missing backbone density. The backrub protocol consisted of repeatedly selecting C_α_ atoms of two residues (separated by 1–10 intervening residues), performing a rigid body rotation of the selected protein segment (of up to 40 degrees), optimizing the location of related C_β_ and hydrogen atoms and accepting or rejecting the backbone move based on the Rosetta energy function and the Monte Carlo Metropolis criterion. Using the atomic coordinates of each crystallographic structure above as a starting conformation, 100 independent backrub simulations were run at two separate Monte Carlo temperatures (kT = 0.6 and kT = 1.2) for 10,000 moves per simulation. At each temperature, the lowest energy conformation sampled as well as the last conformation accepted during each simulation were saved and used to generate a computational ensemble of 400 backbone conformations per starting crystallographic structure.

The conformational fluctuation within the generated backrub ensembles, compared to the ensemble of experimentally determined structures, is shown in **Figure S10**. For two structures of HIV-1 protease (1A8G and 1IZH), ensembles of varying sizes (50 to 1000 structures consisting of last or low energy conformations randomly selected from simulations run at the two Monte Carlo temperatures given above) were systematically tested. It was determined that ensemble sizes of 100 structures or greater gave essentially identical results to the ensemble of experimentally determined protease structures with respect to the area under the curve.

### Evaluation of Receiver Operating Characteristic (ROC) curves and area under the curve (AUC) values

ROC curves were computed for each of 1,728 (protease) and 5,904 (reverse transcriptase) possible mutations (18 amino acid types, excluding the native amino acid residue and cysteine, allowable at 96 sites in protease and 328 sites in reverse transcriptase). Residues that were omitted from the ROC analysis are given above. True positive rates (TPR) and false positive rates (FPR) of mutation recovery were calculated by using the parameter values determined above for the “neutral" and “selective" computational models (**Table S4**) and considering all mutations computationally predicted to occur at frequencies greater than or equal to varying model cutoff values of 30% to 0.00001%. True positives were defined as mutations occurring within the database at >1%. AUC values were calculated for each ROC curve by implementing the trapezoid method.

TPR and FPR rates were also calculated for the set of mutations one nucleotide mutation away from the native codon (**Table S3**) and for the set of all mutations to amino acid types chemically similar to the native amino acid type (chemically similar groupings were as follows: (A,G,P), (D,E,N,Q), (F,W,Y), (L,I,V,M), (R,K,H), (S,T).

## Supporting Information

Figure S1
**Predicted and observed HIV-1 protease amino acid substitutions under neutral conditions.** Amino acid mutations predicted as tolerated by the neutral model (3^rd^ column) are compared with mutations observed in the HIV database with no protease inhibitor treatment (5^th^ column) and functional mutations from mis-sense mutagenesis are also shown (6^th^ column). Model predictions matching amino acid types observed in either the database sequences or the set of experimentally characterized functional point-mutants are shown in bold, red typeface. Residue types unlikely to be predicted by the neutral model, as they are greater than one nucleotide mutation away from the native residue type (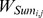 = 0), are shown in blue. Superscripts show predicted and observed frequencies (not available from the mis-sense mutagenesis experiment) rounded to the nearest 1%. The 2^nd^ and 4^th^ columns give overall predicted and observed mutational tolerances for each site, as depicted in **Figure 2**, using the same color-coding. Black triangles in the first column denote residues excluded from the analysis (the catalytic D25 and two cysteine residues).(TIF)

Figure S2 **Burial distribution of background mutations and DRMs in protease.** All protease positions were binned into three groups; buried, intermediate and surface, based on the number of neighboring residues (two residues are neighbors if their C_β_ atoms are within 8 Å; the thresholds of the neighbor number, *n*, for bin assignment were *n*≤10, 10<*n*<13 and *n*≥13 for surface, medium and buried bins, respectively). The graph shows the percent of residues in each burial group for all positions that are involved in DRMs (black bars) or for any possible protease mis-sense mutation reachable by a single nucleotide change (grey bars).
(TIF)

Figure S3 **Comparison of predicted mutational frequencies with and without backbone flexibility for all mutations contained in crystal structures.** For each of 67 mutations contained in at least one crystallographic structure, the frequency with which the mutation was observed in the Stanford database after protease inhibitor treatment (red bars) is compared with the mutational frequency predicted by the selective model using the experimentally determined ensemble (black bars). For comparison, the mutational frequency calculated when using 11 fixed backbone structures crystallized in the absence of mutation (grey bars) as well as the 11 backrub ensembles generated from each fixed backbone structure (striped bars) are also shown. Error bars for the grey and striped bars represent the maximum and minimum mutational frequency values observed over calculations made on the 11 fixed, single crystallographic structures and their computationally generated ensembles, respectively.
(TIF)

Figure S4 **Predicted and observed HIV-1 reverse transcriptase amino acid substitutions for the neutral model.** Data format is as described in the legend to **Figure S1**. Residues not considered in the analysis of the predictions (black triangles) are described in **Figure S9**.
(PDF)

Figure S5 **Predicted and observed HIV-1 reverse transcriptase amino acid substitutions for the selective model.** Data format is as described in the legend to **Figure S1**. Residues not considered in the analysis of the predictions (black triangles) are described in **Figure S9**.
(PDF)

Figure S6 **Spatial distribution of drug resistance mutations in protease and reverse transcriptase.** (**A**) The two protease chains are shown in blue and green colored backbones. Sites of literature-documented DRMs (major and minor; taken [23]) are in red color, showing consensus sequence side chains in stick representation. (**B**) Reverse transcriptase DRMs: literature-documented DRMs (see **Figure 7D** in the main text) are shown as in (**A**). Only residues 1–399 in chains A and B are displayed.
(TIF)

Figure S7 **Improved predictions for a finite set of double mutations.** Secondary mutations that are present in the Stanford database and whose predicted mutational frequencies changed >0.5% (1st column) in the presence of one modeled initial mutations (2nd column). Modeled mutation frequencies in the absence and presence of the initial mutation are given in the 3rd and 4th columns, respectively. For comparison, mutational frequencies observed within the Stanford database after protease inhibitor treatment are listed in the 5th column.
(TIF)

Figure S8 **Structural variability in experimental (A) and computationally generated (B) ensembles of protease, and experimental (C) and computationally generated (D) ensembles of reverse transcriptase.** Structural variability of each ensemble was calculated as described in [19] using mean C_α_ difference distance values of the ensembles. All values were normalized according to the maximum value for each of the four ensembles and color-coded from yellow (less variable) to purple (most variable) as depicted in the legend. Protein segments that were not evaluated due to missing densities in >20 percent of the ensemble members are shown in grey.
(TIF)

Figure S9 **Reverse transcriptase consensus sequence, indicating positions removed from analysis of mutational tolerance.** The reverse transcriptase consensus sequence, extracted from the Stanford HIV database, is colored to represent the groups of residues that were excluded from the analysis: (i) Cysteines (2 positions; green and underline), (ii) Active site (3 positions, highlighted in yellow), (iii) Residues with insufficient information on mutational tolerance in the Stanford HIV database (161 positions, shaded in grey); for these positions, there were only sequences from less than 500 isolates, compared with 1,500–12,100 isolates for all other positions, (iv) DNA/RNA binding - positions that involve DNA/RNA binding in any of the reverse transcriptase-DNA/RNA complexes (46 positions; red), and (v) Regions of missing density in >20% of the available experimentally determined structures (20 positions, blue).
(TIF)

Figure S10 **Comparison of structural variability of the experimentally determined and backrub ensembles for protease.** C_α_ RMS fluctuations for the 263 experimental structures used to calculate *ERES_Fold_* and *ERES_Dimer_* scores and the 16 crystallographic structures used to calculate *ERES_Peptide_* scores are shown in black and red, respectively. For comparison, C_α_ RMS fluctuations for ensembles of structures independently generated by using the backrub protocol (as described in Methods) starting from one of 11 crystallographic structures with the native subtype-B consensus sequences are shown in grey. RMS fluctuations are similar among all ensembles for most of the 99 residues in both chain A (upper graph) and chain B (lower graph) of HIV-protease. Note, the experimentally determined structures show large variation in the flap region (near residue 50) for both chains, as some structures have been solved in the “flap open" conformation. The peptide bound structures show an asymmetric behavior between the two chains for this region while the backrub structures (all generated from a “flap-closed" starting conformation) show smaller fluctuations in the flap region for both chains. The positions of residue 1 and residue 99 are fixed during the backrub protocol, and thus show RMS fluctuations of zero. Each ensemble had an average C_α_ RMSD of 0.2 to 0.6 Å to the original starting template.
(TIF)

Table S1 **PDB codes for the protease and reverse transcriptase structures used for the analysis.** For protease, 263 members of the ensemble of experimentally determined structures used for fold and dimer stability calculations (*ERES_Fold_* and *ERES_Dimer_* scores) are listed. Structures were selected as follows: 386 structures of HIV-1 protease were obtained from the protein databank (PDB) by using the ‘search by sequence’ feature (Blast E-value 0.001) to retrieve structures with sequences similar to 1PRO.pdb (chain A). Structures which contained more than 12 mutations from the HIV-1 subtype B consensus sequence defined above, contained only one chain of the HIV-1 dimer, or had cysteine residues replaced (heteroatom residue codes ABA, CME, CSO, or DBU) were eliminated. Structures determined to be either HIV-2 protease, SIV, Rous sarcoma virus, or tethered dimeric HIV-1 were also eliminated. 262 dimeric HIV-1 crystal structures and one NMR minimized model (PDB code: 1BVG) remained. The experimental ensemble of reverse transcriptase contained 91 structures that have a crystallographic resolution within the range of 1.8 to 3.2 Angstroms. To select these structures, we used the consensus sequence (see **Figure S9**) as a BLAST query against the PDB database. We filtered the results to include structures that are HIV-1 reverse transcriptases (total of 101 structures were found), and excluded structures with >24 deviations from the consensus sequence.
(TIF)

Table S2 **PDB codes and substrate peptides of the structures used for peptide substrate binding calculations (** ***ERES_Peptide_*** **scores).** For each of the 10 endogenous peptides considered, the PDB codes of all crystal structures used, as well as their peptide sequence present in the crystallographic structure, are given. All peptides are denoted from P5–P5' except for 1TSQ, 2FNS, and 2FNT which are given from P4–P6'. Amino acids not in the crystal structure (and thus not present in computational simulations) are shown in grey. Amino acids colored red are peptide mutations observed in response to the HIV-1 protease drug resistance mutation V82A. Amino acids depicted in blue were computationally engineered for tighter protease binding affinity.
(TIF)

Table S3
**Cases in which** 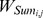 **was set to zero due to mutations that require more than a single nucleotide change.** Rows: amino acid type in the consensus sequence; columns: amino acid types reachable (value of one) or not reachable (empty box) by a single nucleotide change. Boxes colored blue denote mutations considered to be tolerated for a null model of chemically similar amino acid types. Leucines and arginines were assigned to one of two codons as follows: L1 (residues 5, 10, 19, 23, 63, 76, 89) or L2 (residues 24, 33, 38, 90, 97); R1 (residue 8) or R2 (residues 41, 57, 87). There were no serines in the HIV-1 protease sequence to assign to S1 or S2. The assignments above matched the Stanford database, except for mis-assignment of the codon for 76L to L1 instead of L2. For reverse transcriptase, L1 was assigned to positions (80, 109, 149, 205, 209, 234, 246, 282, 283, 295, 301, 303, 310, 349, 391), L2 was assigned to positions (12, 34, 100, 120, 168, 187, 193, 210, 260, 264, 279, 325, 368), R2 was assigned to positions (72, 83, 125, 143, 172, 199, 206, 211, 307, 356), S1 was assigned to positions (48, 105, 117, 156, 191, 322) and S2 was assigned to positions (68, 134, 162, 163, 251, 268, 379]. There were no Arginines in the reverse transcriptase sequence to assign to R1.(TIF)

Table S4 **Parameter values used in the neutral and selective computational models.** For parameter optimization, all combinations of the parameter values listed were tested for their predictive ability in determining overall mutational frequencies at each of the 96 sequence sites in HIV-1 protease averaged over 3 bins, as described in Methods.
(TIF)

Table S5 **Predicted stabilization/destabilization effect of frequent major DRM combinations.** Frequencies of HIV sequences that include mutation combinations with DRMs that are found in >50 sequences were obtained from the Stanford HIV database (http://hivdb.stanford.edu/pages/phenoSummary/Pheno.PI.Simple.html). The 1^st^ and 2^nd^ columns list the mutation combinations and sequence frequencies, respectively. Rosetta scores for these DRMs were assigned with wither “−" or “+" signs (for stabilizing (*ERES_Fold_*<0) or destabilizing effects (*ERES_Fold_*>0), respectively). DRMs for which our model did not provide predictions are given “?" signs. The 4^th^ column is assigned with a positive sign in cases where a combination of predicted stabilizing and destabilizing mutations are found (corresponding to a compensation scenario). Negative signs are assigned where this is not the case (a “?" sign denotes the single case in which the compensating/non-compensating scenario could not be determined).
(PDF)

Text S1 **Additional details of computational methods.** Description of the Rosetta energy terms, the command line for Rosetta backrub simulations, computational time requirements and simulations to estimate the effect of correlated mutations.
(PDF)
