# Supplementary figures and images for: Prediction of Mutational Tolerance in HIV-1 Protease and Reverse Transcriptase Using Flexible Backbone Protein Design
Source: PLoS Comput Biol. 2012 Aug 23;8(8):e1002639. doi: 10.1371/journal.pcbi.1002639 (PMC3426558; doi:10.1371/journal.pcbi.1002639)

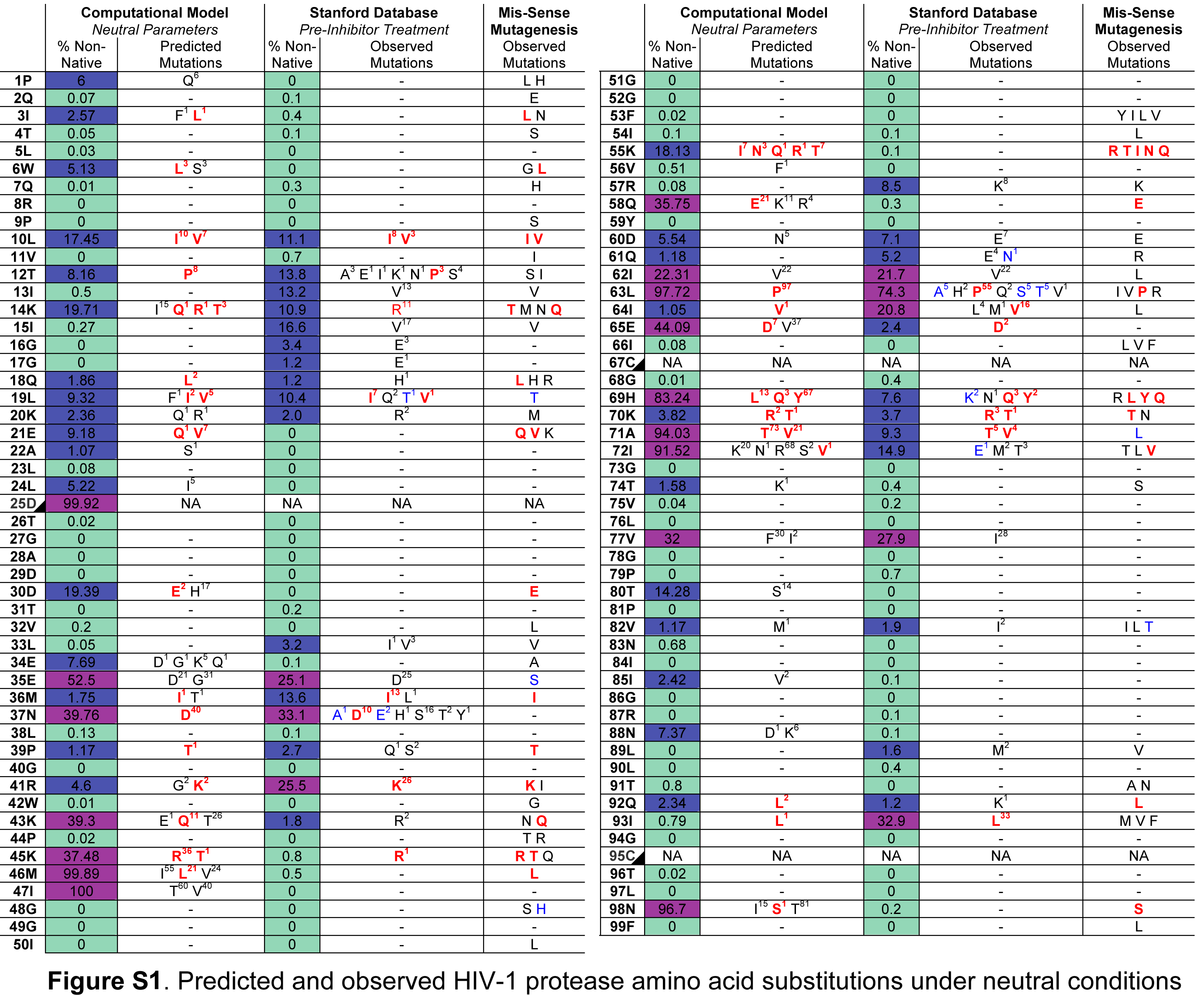

Supplement: Figure S1 — Predicted and observed HIV-1 protease amino acid substitutions under neutral conditions. Amino acid mutations predicted as tolerated by the neutral model (3rd column) are compared with mutations observed in the HIV database with no protease inhibitor treatment (5th column) and functional mutations from mis-sense mutagenesis are also shown (6th column). Model predictions matching amino acid types observed in either the database sequences or the set of experimentally characterized functional point-mutants are shown in bold, red typeface. Residue types unlikely to be predicted by the neutral model, as they are greater than one nucleotide mutation away from the native residue type ( = 0), are shown in blue. Superscripts show predicted and observed frequencies (not available from the mis-sense mutagenesis experiment) rounded to the nearest 1%. The 2nd and 4th columns give overall predicted and observed mutational tolerances for each site, as depicted in Figure 2 , using the same color-coding. Black triangles in the first column denote residues excluded from the analysis (the catalytic D25 and two cysteine residues). (TIF) [file pcbi.1002639.s001.tif]

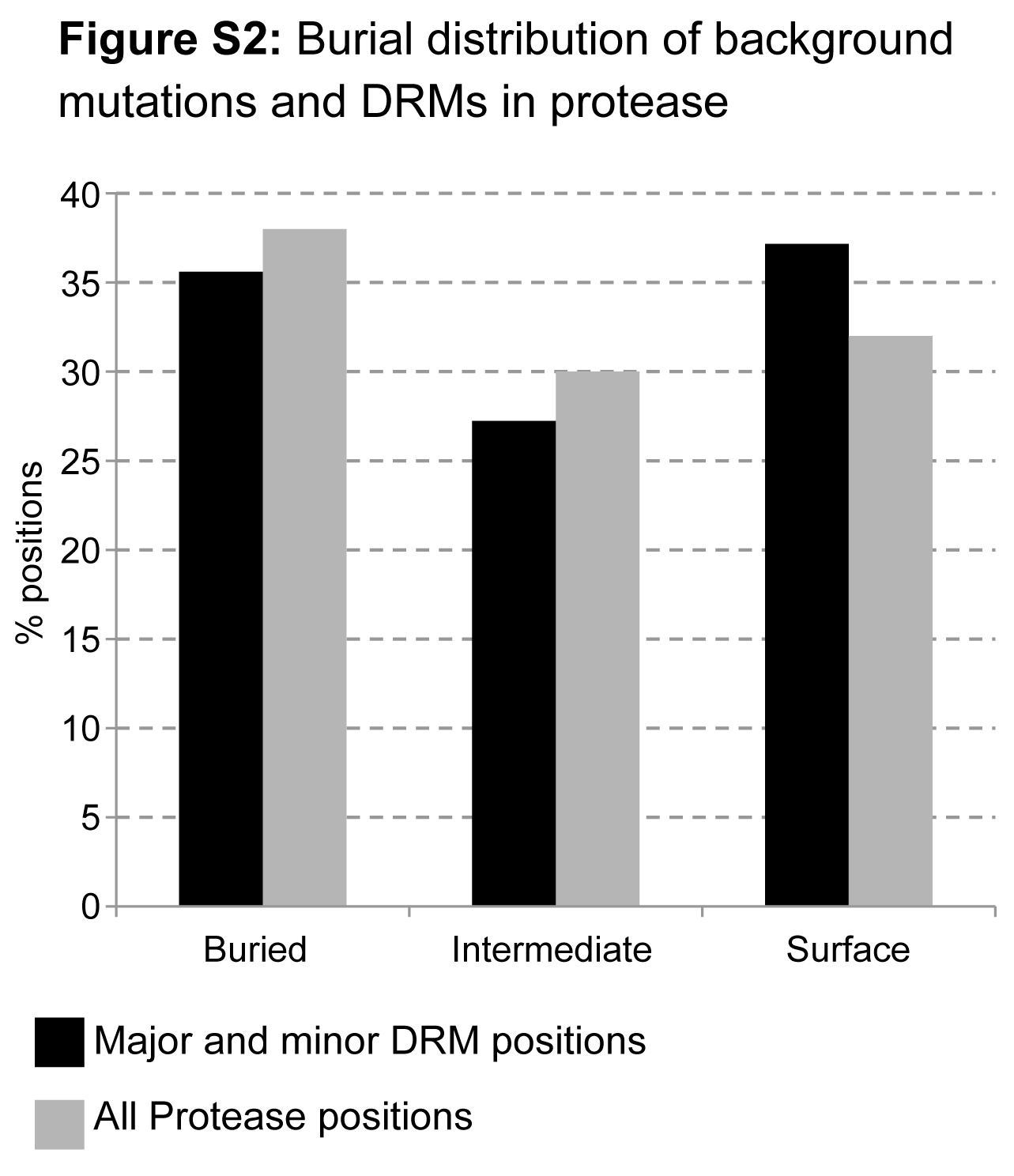

Supplement: Figure S2 — Burial distribution of background mutations and DRMs in protease. All protease positions were binned into three groups; buried, intermediate and surface, based on the number of neighboring residues (two residues are neighbors if their Cβ atoms are within 8 Å; the thresholds of the neighbor number, n, for bin assignment were n≤10, 10<n<13 and n≥13 for surface, medium and buried bins, respectively). The graph shows the percent of residues in each burial group for all positions that are involved in DRMs (black bars) or for any possible protease mis-sense mutation reachable by a single nucleotide change (grey bars). (TIF) [file pcbi.1002639.s002.tif]

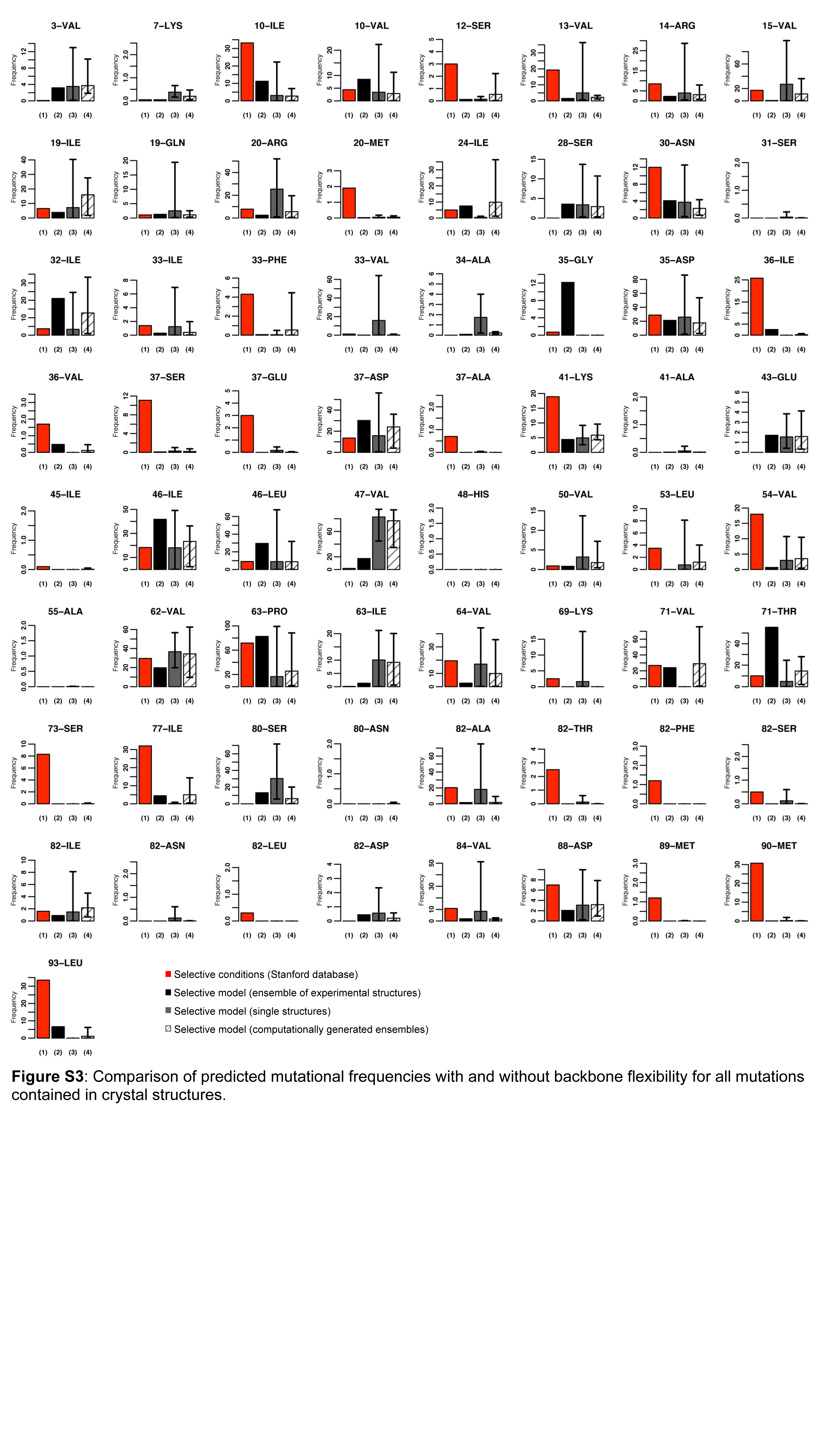

Supplement: Figure S3 — Comparison of predicted mutational frequencies with and without backbone flexibility for all mutations contained in crystal structures. For each of 67 mutations contained in at least one crystallographic structure, the frequency with which the mutation was observed in the Stanford database after protease inhibitor treatment (red bars) is compared with the mutational frequency predicted by the selective model using the experimentally determined ensemble (black bars). For comparison, the mutational frequency calculated when using 11 fixed backbone structures crystallized in the absence of mutation (grey bars) as well as the 11 backrub ensembles generated from each fixed backbone structure (striped bars) are also shown. Error bars for the grey and striped bars represent the maximum and minimum mutational frequency values observed over calculations made on the 11 fixed, single crystallographic structures and their computationally generated ensembles, respectively. (TIF) [file pcbi.1002639.s003.tif]

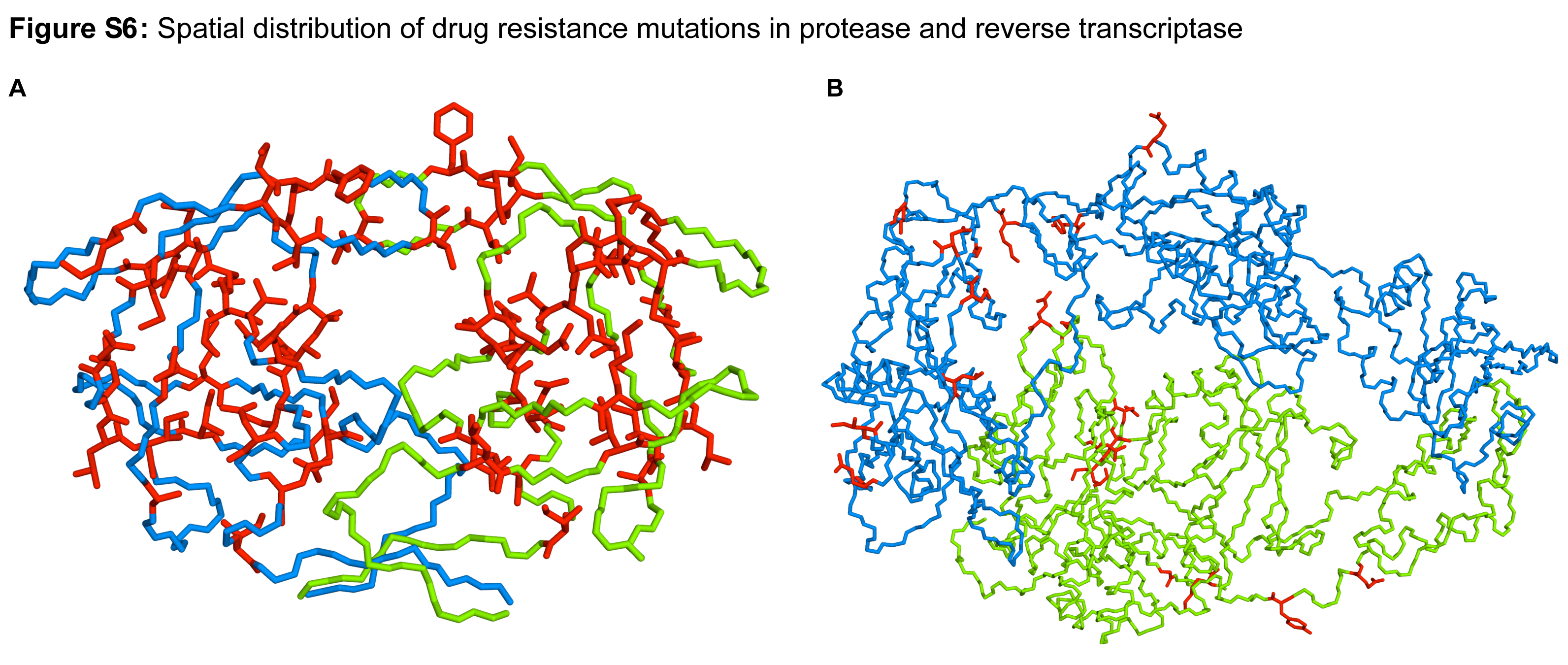

Supplement: Figure S6 — Spatial distribution of drug resistance mutations in protease and reverse transcriptase. (A) The two protease chains are shown in blue and green colored backbones. Sites of literature-documented DRMs (major and minor; taken [23]) are in red color, showing consensus sequence side chains in stick representation. (B) Reverse transcriptase DRMs: literature-documented DRMs (see Figure 7D in the main text) are shown as in (A). Only residues 1–399 in chains A and B are displayed. (TIF) [file pcbi.1002639.s006.tif]

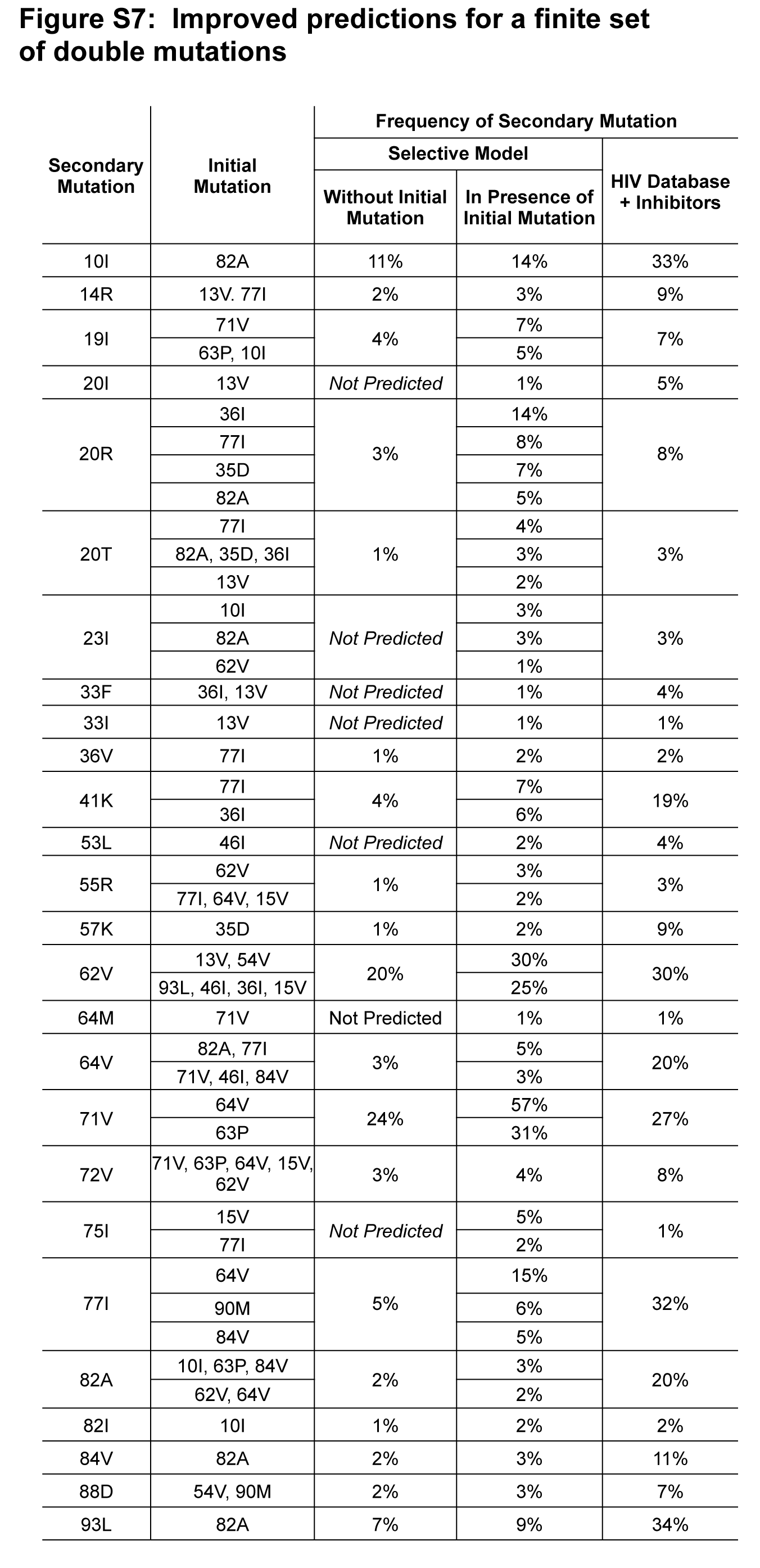

Supplement: Figure S7 — Improved predictions for a finite set of double mutations. Secondary mutations that are present in the Stanford database and whose predicted mutational frequencies changed >0.5% (1st column) in the presence of one modeled initial mutations (2nd column). Modeled mutation frequencies in the absence and presence of the initial mutation are given in the 3rd and 4th columns, respectively. For comparison, mutational frequencies observed within the Stanford database after protease inhibitor treatment are listed in the 5th column. (TIF) [file pcbi.1002639.s007.tif]

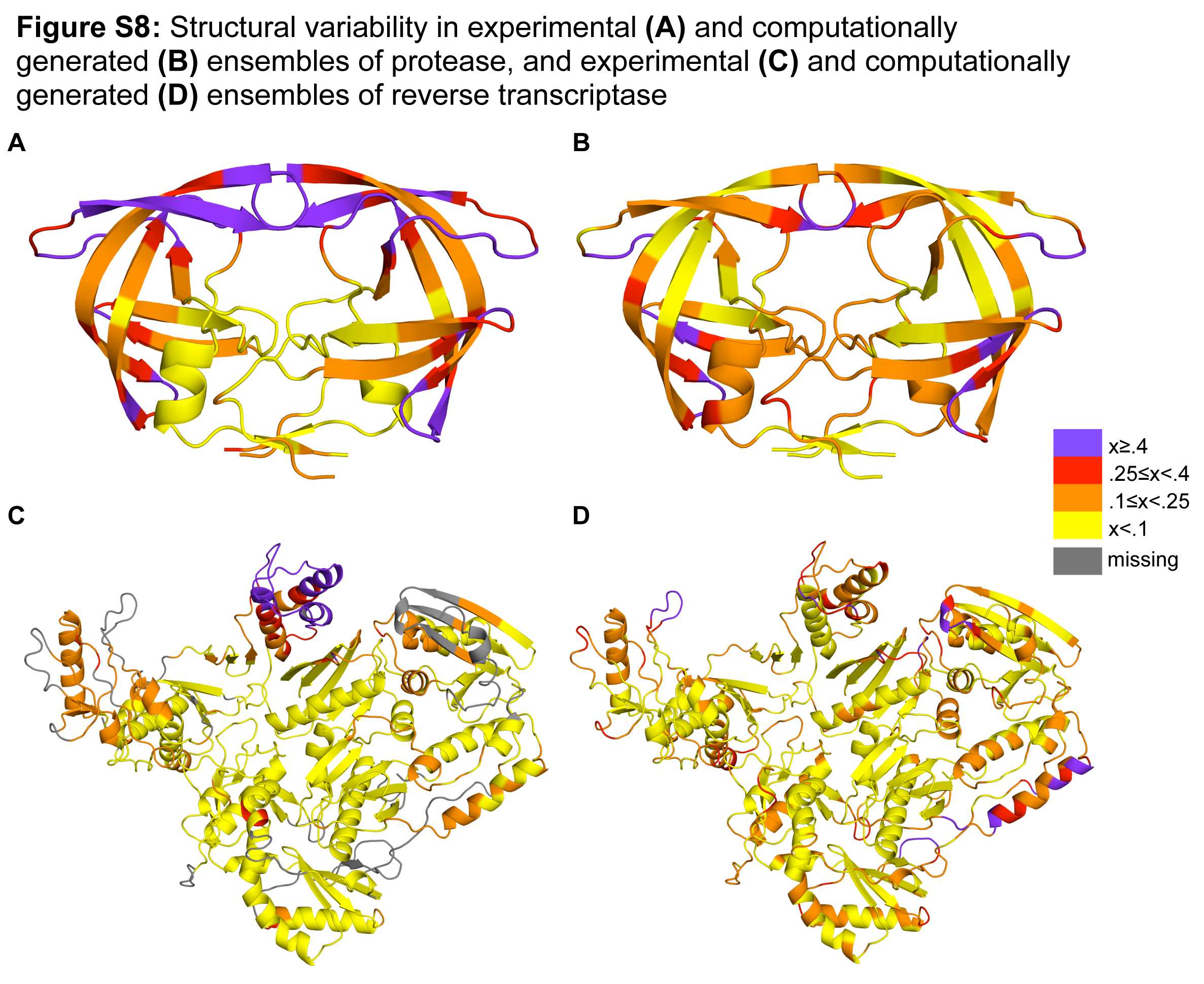

Supplement: Figure S8 — Structural variability in experimental (A) and computationally generated (B) ensembles of protease, and experimental (C) and computationally generated (D) ensembles of reverse transcriptase. Structural variability of each ensemble was calculated as described in [19] using mean Cα difference distance values of the ensembles. All values were normalized according to the maximum value for each of the four ensembles and color-coded from yellow (less variable) to purple (most variable) as depicted in the legend. Protein segments that were not evaluated due to missing densities in >20 percent of the ensemble members are shown in grey. (TIF) [file pcbi.1002639.s008.tif]

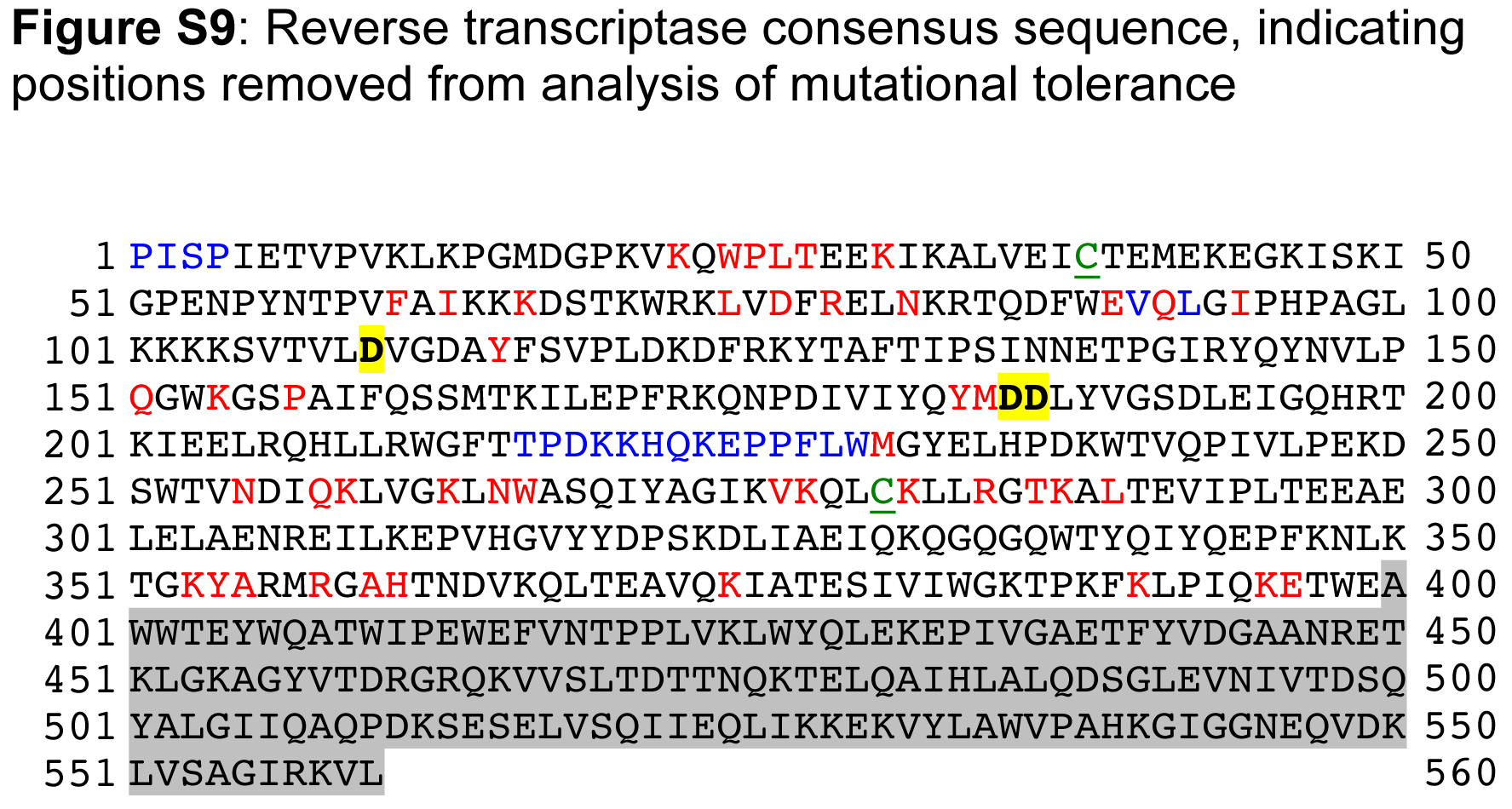

Supplement: Figure S9 — Reverse transcriptase consensus sequence, indicating positions removed from analysis of mutational tolerance. The reverse transcriptase consensus sequence, extracted from the Stanford HIV database, is colored to represent the groups of residues that were excluded from the analysis: (i) Cysteines (2 positions; green and underline), (ii) Active site (3 positions, highlighted in yellow), (iii) Residues with insufficient information on mutational tolerance in the Stanford HIV database (161 positions, shaded in grey); for these positions, there were only sequences from less than 500 isolates, compared with 1,500–12,100 isolates for all other positions, (iv) DNA/RNA binding - positions that involve DNA/RNA binding in any of the reverse transcriptase-DNA/RNA complexes (46 positions; red), and (v) Regions of missing density in >20% of the available experimentally determined structures (20 positions, blue). (TIF) [file pcbi.1002639.s009.tif]

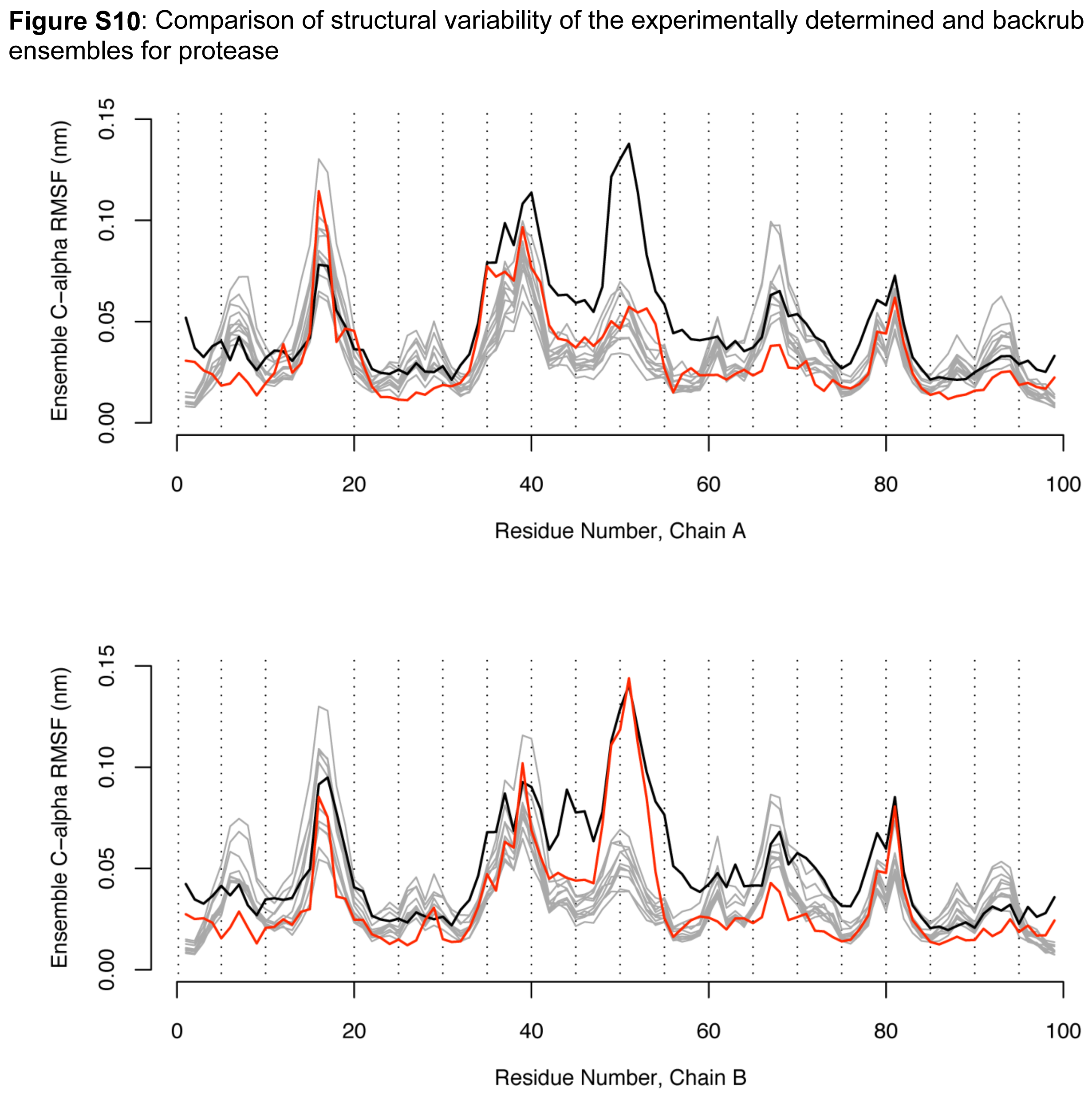

Supplement: Figure S10 — Comparison of structural variability of the experimentally determined and backrub ensembles for protease. Cα RMS fluctuations for the 263 experimental structures used to calculate ERESFold and ERESDimer scores and the 16 crystallographic structures used to calculate ERESPeptide scores are shown in black and red, respectively. For comparison, Cα RMS fluctuations for ensembles of structures independently generated by using the backrub protocol (as described in Methods) starting from one of 11 crystallographic structures with the native subtype-B consensus sequences are shown in grey. RMS fluctuations are similar among all ensembles for most of the 99 residues in both chain A (upper graph) and chain B (lower graph) of HIV-protease. Note, the experimentally determined structures show large variation in the flap region (near residue 50) for both chains, as some structures have been solved in the “flap open" conformation. The peptide bound structures show an asymmetric behavior between the two chains for this region while the backrub structures (all generated from a “flap-closed" starting conformation) show smaller fluctuations in the flap region for both chains. The positions of residue 1 and residue 99 are fixed during the backrub protocol, and thus show RMS fluctuations of zero. Each ensemble had an average Cα RMSD of 0.2 to 0.6 Å to the original starting template. (TIF) [file pcbi.1002639.s010.tif]

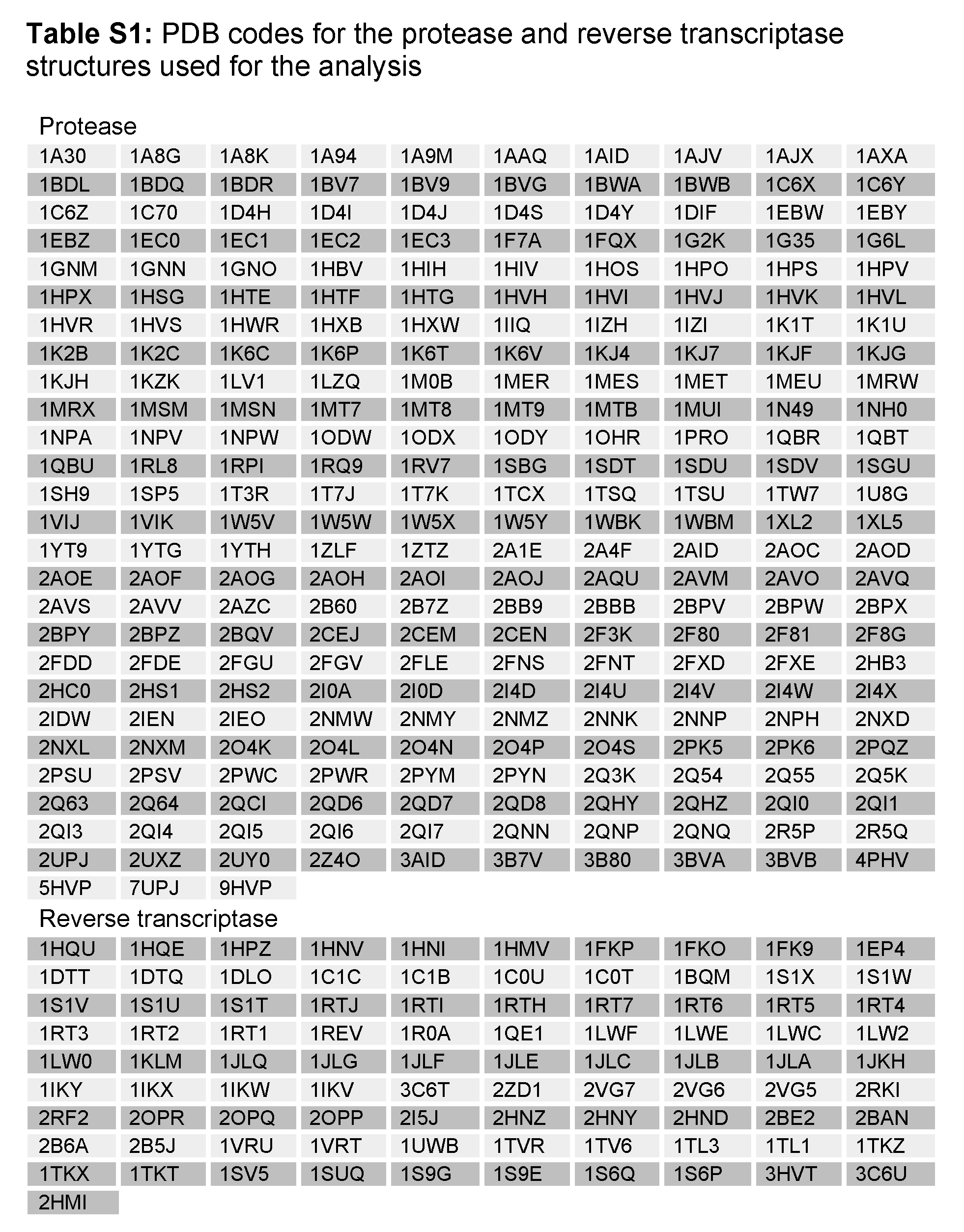

Supplement: Table S1 — PDB codes for the protease and reverse transcriptase structures used for the analysis. For protease, 263 members of the ensemble of experimentally determined structures used for fold and dimer stability calculations (ERESFold and ERESDimer scores) are listed. Structures were selected as follows: 386 structures of HIV-1 protease were obtained from the protein databank (PDB) by using the ‘search by sequence’ feature (Blast E-value 0.001) to retrieve structures with sequences similar to 1PRO.pdb (chain A). Structures which contained more than 12 mutations from the HIV-1 subtype B consensus sequence defined above, contained only one chain of the HIV-1 dimer, or had cysteine residues replaced (heteroatom residue codes ABA, CME, CSO, or DBU) were eliminated. Structures determined to be either HIV-2 protease, SIV, Rous sarcoma virus, or tethered dimeric HIV-1 were also eliminated. 262 dimeric HIV-1 crystal structures and one NMR minimized model (PDB code: 1BVG) remained. The experimental ensemble of reverse transcriptase contained 91 structures that have a crystallographic resolution within the range of 1.8 to 3.2 Angstroms. To select these structures, we used the consensus sequence (see Figure S9) as a BLAST query against the PDB database. We filtered the results to include structures that are HIV-1 reverse transcriptases (total of 101 structures were found), and excluded structures with >24 deviations from the consensus sequence. (TIF) [file pcbi.1002639.s011.tif]

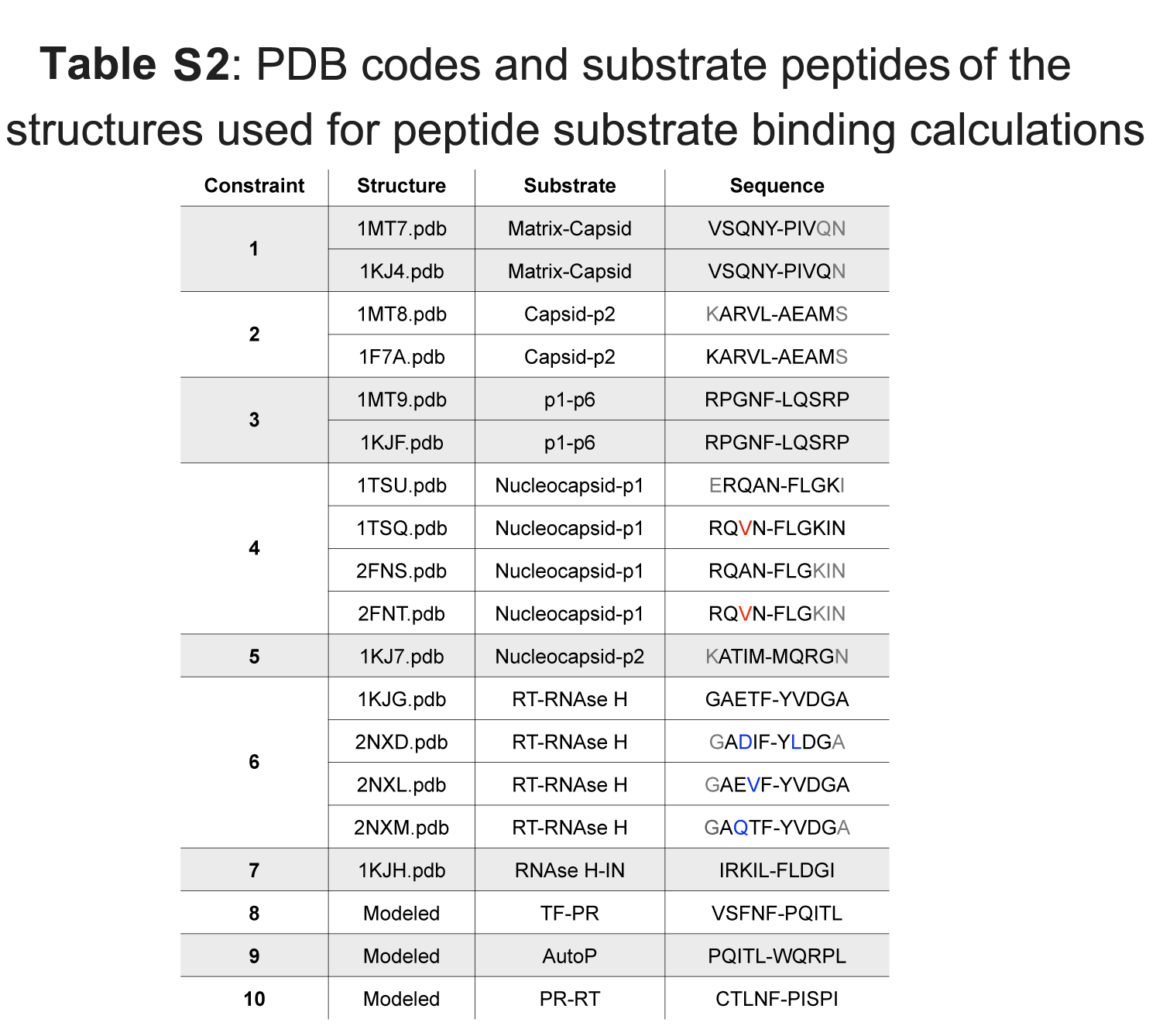

Supplement: Table S2 — PDB codes and substrate peptides of the structures used for peptide substrate binding calculations ( ERESPeptide scores). For each of the 10 endogenous peptides considered, the PDB codes of all crystal structures used, as well as their peptide sequence present in the crystallographic structure, are given. All peptides are denoted from P5–P5' except for 1TSQ, 2FNS, and 2FNT which are given from P4–P6'. Amino acids not in the crystal structure (and thus not present in computational simulations) are shown in grey. Amino acids colored red are peptide mutations observed in response to the HIV-1 protease drug resistance mutation V82A. Amino acids depicted in blue were computationally engineered for tighter protease binding affinity. (TIF) [file pcbi.1002639.s012.tif]

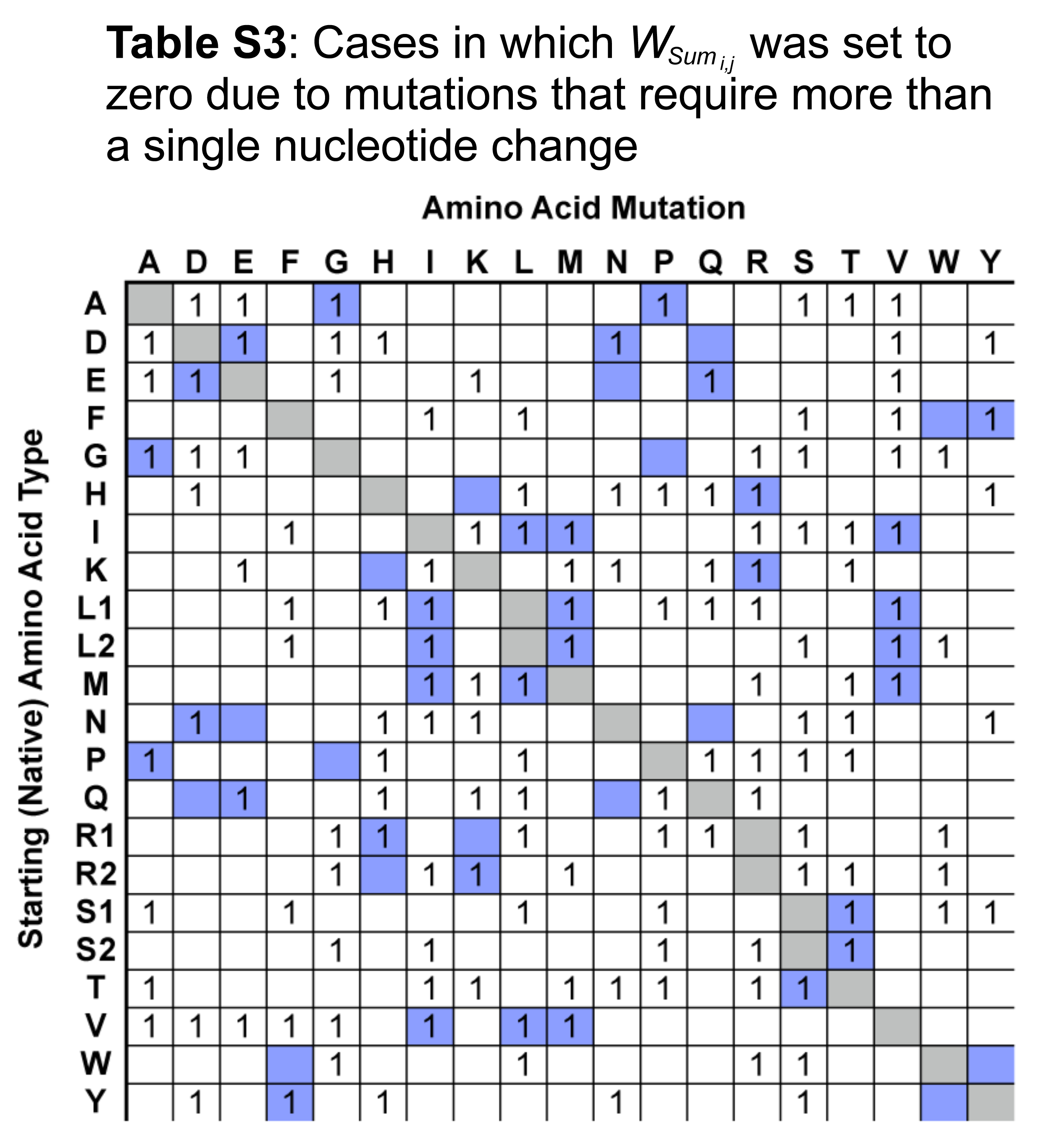

Supplement: Table S3 — Cases in which was set to zero due to mutations that require more than a single nucleotide change. Rows: amino acid type in the consensus sequence; columns: amino acid types reachable (value of one) or not reachable (empty box) by a single nucleotide change. Boxes colored blue denote mutations considered to be tolerated for a null model of chemically similar amino acid types. Leucines and arginines were assigned to one of two codons as follows: L1 (residues 5, 10, 19, 23, 63, 76, 89) or L2 (residues 24, 33, 38, 90, 97); R1 (residue 8) or R2 (residues 41, 57, 87). There were no serines in the HIV-1 protease sequence to assign to S1 or S2. The assignments above matched the Stanford database, except for mis-assignment of the codon for 76L to L1 instead of L2. For reverse transcriptase, L1 was assigned to positions (80, 109, 149, 205, 209, 234, 246, 282, 283, 295, 301, 303, 310, 349, 391), L2 was assigned to positions (12, 34, 100, 120, 168, 187, 193, 210, 260, 264, 279, 325, 368), R2 was assigned to positions (72, 83, 125, 143, 172, 199, 206, 211, 307, 356), S1 was assigned to positions (48, 105, 117, 156, 191, 322) and S2 was assigned to positions (68, 134, 162, 163, 251, 268, 379]. There were no Arginines in the reverse transcriptase sequence to assign to R1. (TIF) [file pcbi.1002639.s013.tif]

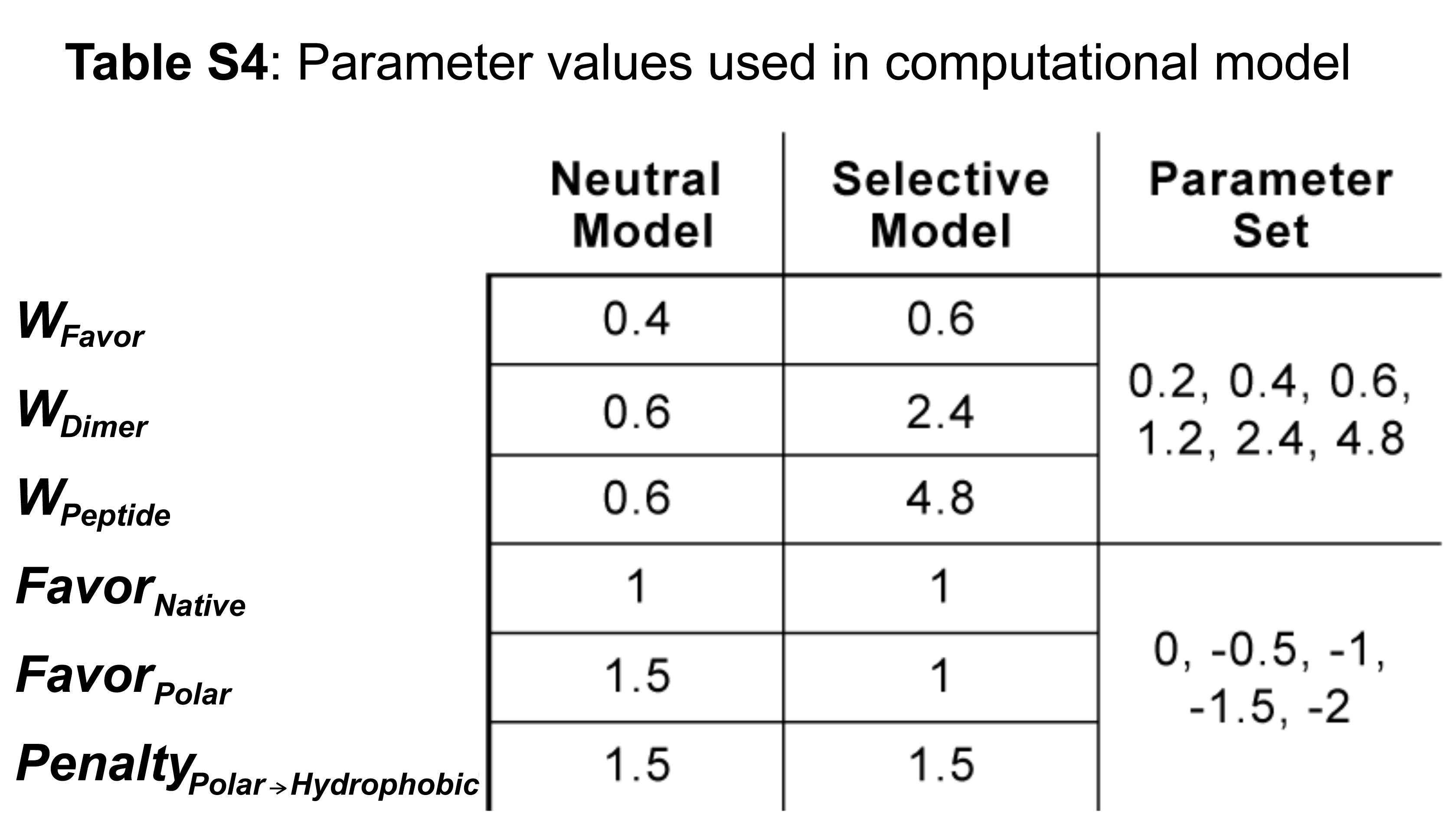

Supplement: Table S4 — Parameter values used in the neutral and selective computational models. For parameter optimization, all combinations of the parameter values listed were tested for their predictive ability in determining overall mutational frequencies at each of the 96 sequence sites in HIV-1 protease averaged over 3 bins, as described in Methods. (TIF) [file pcbi.1002639.s014.tif]
