# Supplementary material for: Prediction of Mutational Tolerance in HIV-1 Protease and Reverse Transcriptase Using Flexible Backbone Protein Design
Source: PLoS Comput Biol. 2012 Aug 23;8(8):e1002639. doi: 10.1371/journal.pcbi.1002639 (PMC3426558; doi:10.1371/journal.pcbi.1002639)
Supplement: Figure S4 — Predicted and observed HIV-1 reverse transcriptase amino acid substitutions for the neutral model. Data format is as described in the legend to Figure S1. Residues not considered in the analysis of the predictions (black triangles) are described in Figure S9. (PDF) [file pcbi.1002639.s004.pdf]

Figure S4: Predicted and observed HIV-1 reverse transcriptase amino acid substitutions for the neutral model

	Computational Model *Neutral parameters*		Stanford Database *Pre-inhibitor Treatment*	
	% Non-Native	Predicted Mutations	% Non-Native	Observed Mutations
1P	97.55	L^{13} Q^{69} S^{13} T^{2}	0	-
2I	0.67	V^{1}	0	-
3S	0.02	-	0	-
4P	0	-	0.5	-
5I	15.89	**V^{16}**	0.5	**V^{1}**
6E	96.57	**D^{96}**	7.8	**D^{7}** K^{1}
7T	30.44	P^{30}	0.1	-
8V	0.08	-	0.9	I^{1}
9P	0.94	Q^{1}	0	-
10V	0	-	0	-
11K	100	T^{100}	2.3	R^{2}
12L	0	-	0	-
13K	0	-	0	-
14P	0	-	0	-
15G	0	-	0	-
16M	99.95	L^{16} T^{84}	0	-
17D	0	-	0	-
18G	0	-	0	-
19P	0	-	0	-
20K	91.02	N^{27} **R^{63}**	9.8	**R^{10}**
21V	0.04	-	1.1	I^{1}
22K	6.82	E^{1} Q^{3} R^{1} T^{2}	0.4	-
23Q	0	-	0	-
24W	100	L^{95} R^{3} S^{1}	0	-
25P	0	-	0	-
26L	0	-	0.1	-
27T	5.95	P^{6}	0.5	S^{1}
28E	0	-	0.8	A^{1}
29E	0.09	-	0	-
30K	16.10	**Q^{16}**	0.9	**Q^{1}**
31I	0.01	-	0.2	-
32K	26.09	Q^{1} **R^{24}**	2.4	G^{1} **R^{1}**
33A	0	-	0	-
34L	0	-	0.9	T^{1}
35V	99.39	E^{2} **I^{7}** **L^{90}**	27.4	**I^{15}** **L^{4}** M^{2} T^{6}
36E	1.46	K^{1} Q^{1}	3	D^{1} N^{2}
37I	0.13	-	1.9	F^{2}
38C	NA	NA	NA	NA
39T	86.42	**K^{54}** N^{24} R^{8}	4.6	A^{3} **K^{1}**
40E	4.09	K^{3} Q^{1}	0.5	D^{1}
41M	15.89	**L^{16}**	1.7	**L^{2}**
42E	0	-	0	-
43K	36.22	E^{5} N^{28} Q^{1} R^{2}	0.8	-
44E	51.41	D^{21} K^{9} Q^{21}	0.3	-
45G	0	-	0.1	-
46K	15.89	R^{16}	0.1	-
47I	0.13	-	0	-
48S	0.29	-	2.6	E^{1} T^{2}
49K	1.62	**R^{1}** T^{1}	4.7	**R^{5}**
50I	3.03	T^{2} **V^{2}**	1.4	**V^{1}**

	Computational Model *Neutral parameters*		Stanford Database *Pre-inhibitor Treatment*	
	% Non-Native	Predicted Mutations	% Non-Native	Observed Mutations
51G	0	-	0	-
52P	0.02	-	0	-
53E	100	**D^{100}**	0.6	**D^{1}**
54N	100	D^{97} I^{3}	0	-
55P	0.01	-	0	-
56Y	0	-	0	-
57N	69.71	S^{70}	0	-
58T	1.53	I^{2}	0	-
59P	0	-	0	-
60V	0.29	-	11.6	I^{12}
61F	3.44	Y^{3}	0	-
62A	73.49	S^{12} T^{1} V^{61}	0.2	-
63I	0.13	-	0	-
64K	8.08	N^{8}	1.5	R^{2}
65K	10.50	E^{7} N^{1} Q^{1}	0.1	-
66K	100	N^{100}	0	-
67D	99.53	G^{69} **N^{30}**	0.7	**N^{1}**
68S	96.83	**G^{89}** N^{7} T^{1}	4.2	**G^{4}**
69T	99.98	**N^{100}**	1.3	**N^{1}** S^{1}
70K	99.82	E^{12} Q^{61} T^{27}	0.4	-
71W	0	-	0	-
72R	0	-	0	-
73K	100	E^{2} N^{98}	0	-
74L	3.45	V^{3}	0.1	-
75V	0	-	0	-
76D	0	-	0	-
77F	0	-	0	-
78R	0	-	0	-
79E	0	-	0.2	-
80L	0	-	0	-
81N	1.55	D^{2}	0	-
82K	2.18	N^{2} R^{1}	0.4	-
83R	0	-	16.3	K^{16}
84T	0	-	0	-
85Q	0	-	0	-
86D	0.10		1.1	E^{1}
87F	0	-	0	-
88W	30.30	S^{30}	0	-
89E	46.91	D^{3} K^{3} Q^{40}	0	-
90V	57.07	G^{43} **I^{14}**	1.6	**I^{2}**
91Q	12.55	L^{13}	0	-
92L	0	-	0	-
93G	0	-	0	-
94I	99.65	L^{10} T^{90}	0	-
95P	0	-	0	-
96H	0	-	0	-
97P	0	-	0	-
98A	100	**S^{100}**	6.1	**S^{6}**
99G	0	-	0	-
100L	0	-	0	-

	Computational Model *Neutral parameters*		Stanford Database *Pre-inhibitor Treatment*	
	% Non-Native	Predicted Mutations	% Non-Native	Observed Mutations
101K	0.28	-	1.7	Q^{1} R^{1}
102K	34.75	E^{1} N^{28} **Q^{5}**	5.1	**Q^{3}** R^{2}
103K	5.50	Q^{1} T^{4}	4.9	N^{2} R^{3}
104K	2.29	N^{2} T^{1}	2.5	R^{2}
105S	88.37	F^{62} Y^{27}	0.2	-
106V	0	-	1.7	I^{2}
107T	0	-	0.2	-
108V	0	-	0.3	-
109L	0	-	0	-
110D	NA	NA	NA	NA
111V	96.56	I^{97}	0.3	-
112G	31.07	A^{1} S^{30}	0	-
113D	69.72	N^{70}	0	-
114A	0	-	0	-
115Y	0.02	-	0	-
116F	99.94	L^{100}	0	-
117S	5.45	L^{1} T^{3}	0	-
118V	0	-	2.6	I^{3}
119P	0	-	0	-
120L	0	-	0	-
121D	0.13	-	5	H^{3} Y^{2}
122K	37.48	**E^{27} Q^{5}** R^{5}	31.9	**E^{27}** P^{3} **Q^{1}**
123D	3.01	**E^{2} N^{2}**	28.9	**E^{24}** G^{1} **N^{3}** S^{1}
124F	50.00	Y^{50}	0	-
125R	0	-	0	-
126K	0.55	E^{1}	0	-
127Y	15.89	F^{16}	0	-
128T	0	-	0	-
129A	0	-	0	-
130F	0	-	0	-
131T	0	-	0	-
132I	0.01	-	0.1	-
133P	0	-	0	-
134S	0	-	0	-
135I	58.52	K^{2} **L^{18} R^{3}** S^{2} **T^{3} V^{31}**	42.8	**L^{3}** M^{1} **R^{3} T^{28} V^{8}**
136N	0	-	0	-
137N	25.05	H^{25}	0	-
138E	12.74	D^{12}	2.1	A^{2}
139T	0.01	-	0.8	-
140P	0.16	-	0	-
141G	0	-	0	-
142I	6.20	K^{4} **V^{1}**	9.4	T^{3} **V^{6}**
143R	1.69	I^{2}	0.1	-
144Y	0	-	0	-
145Q	92.42	R^{92}	0	-
146Y	0.29		0	-
147N	0.67	D^{1}	0	-
148V	0	-	0	-
149L	0	-	0	-
150P	0	-	0	-

	Computational Model *Neutral parameters*		Stanford Database *Pre-inhibitor Treatment*	
	% Non-Native	Predicted Mutations	% Non-Native	Observed Mutations
151Q	99.87	P^{100}	0	-
152G	0	-	0	-
153W	0	-	0	-
154K	0	-	0	-
155G	0	-	0	-
156S	99.87	A^{100}	0	-
157P	1.80	A^{2}	0	-
158A	0.01	-	1.7	S^{2}
159I	0	-	0.2	-
160F	0	-	0	-
161Q	6.70	E^{3} L^{3} R^{1}	0	-
162S	33.36	R^{4} T^{29}	6.5	A^{4} Y^{2}
163S	17.43	I^{1} N^{1} T^{16}	0.1	-
164M	100	L^{100}	0	-
165T	13.98	K^{9} R^{4}	3	I^{3}
166K	0.52	-	7.2	R^{7}
167I	0.02	-	0	-
168L	0	-	0	-
169E	0	-	5	D^{5}
170P	98.54	L^{1} Q^{94} R^{3}	0	-
171F	0	-	0.6	Y^{1}
172R	1.70	I^{2}	0.2	-
173K	2.28	**N^{1} Q^{2}**	7.4	A^{1} E^{3} **N^{1} Q^{1}** R^{1} T^{1}
174Q	1.74	**E^{1} K^{1}**	7.2	**E^{2}** H^{1} **K^{3}** R^{1}
175N	15.93	**H^{16}**	1	**H^{1}**
176P	0.12	-	0.5	-
177D	0.06	-	21.4	E^{20} G^{1} N^{1}
178I	15.89	**V^{16}**	15.5	L^{5} M^{9} **V^{1}**
179V	0.10	-	6.1	D^{2} I^{4}
180I	0.39	-	0.7	V^{1}
181Y	0.07	-	0	-
182Q	94.15	L^{94}	0	-
183Y	50.01	F^{50}	0	-
184M	0.15	-	1	V^{1}
185D	NA	NA	NA	NA
186D	NA	NA	NA	NA
187L	0	-	0	-
188Y	69.71	F^{70}	0.1	-
189V	0	-	1	I^{1}
190G	0.30	-	0.2	-
191S	0	-	0	-
192D	0.30	-	0.1	-
193L	7.59	S^{8}	0	-
194E	96.13	D^{47} K^{2} Q^{47}	0.3	-
195I	100	K^{30} **L^{2}** N^{2} R^{30} S^{6} T^{30}	1.3	**L^{1}**
196G	100	D^{2} **E^{98}**	14.9	**E^{14}**
197Q	28.38	**K^{14}** L^{1} R^{14}	3.4	E^{1} **K^{1}**
198H	0	-	0	-
199R	0	-	0	-
200T	92.64	K^{90} N^{1} R^{1}	30.5	A^{18} E^{2} I^{9} V^{1}

	Computational Model *Neutral parameters*		Stanford Database *Pre-inhibitor Treatment*	
	% Non-Native	Predicted Mutations	% Non-Native	Observed Mutations
201K	4.24	N^{3} Q^{1}	0.3	-
202I	100	**V^{100}**	9.4	**V^{9}**
203E	0.96	**D^{1}**	1	**D^{1}**
204E	0.01	-	4	D^{1} K^{2} Q^{1}
205L	0	-	0	-
206R	0.01	-	0	-
207Q	7.81	**E^{8}**	24.7	A^{2} D^{1} **E^{17}** G^{1} H^{1} K^{2} N^{1} R^{1}
208H	30.47	D^{30}	0.2	-
209L	0	-	0	-
210L	0.37	-	1.5	F^{1} W^{1}
211R	0.67	**K^{1}**	52.1	A^{1} G^{4} **K^{43}** Q^{2} S^{2} T^{1}
212W	99.98	R^{99} S^{1}	0	-
213G	0	-	0	-
214F	67.06	I^{33} **L^{33}** V^{1}	14.3	**L^{14}**
215T	2.46	I^{1} R^{2}	2.5	D^{1} S^{1}
216T	0.13	-	0	-
217P	0	-	0	-
218D	0	-	0.1	-
219K	7.60	E^{8}	0.5	-
220K	72.17	N^{64} Q^{2} T^{5}	0	-
221H	100	D^{16} N^{83} P^{1}	0.1	-
222Q	3.45	L^{3}	0	-
223K	99.99	T^{100}	0	-
224E	99.98	D^{30} G^{70}	0.4	-
225P	0	-	0.1	-
226P	0	-	0	-
227F	99.87	Y^{100}	0	-
228L	58.57	I^{8} Q^{41} R^{8} V^{1}	0.3	-
229W	0	-	0	-
230M	43.44	L^{25} R^{19}	0	-
231G	0	-	0	-
232Y	0.13	-	0	-
233E	69.85	D^{69}	0	-
234L	0.01	-	0	-
235H	0.67	Y^{1}	0	-
236P	0	-	0	-
237D	0.07	-	0.2	-
238K	84.55	N^{82} Q^{1} **R^{1}**	0.7	**R^{1}**
239W	0	-	0	-
240T	0.73	S^{1}	0	-
241V	99.71	D^{100}	0.2	-
242Q	0.13	-	0	-
243P	0	-	0.5	-
244I	0.68	**V^{1}**	1.2	**V^{1}**
245V	69.88	D^{69}	38	E^{11} I^{2} K^{6} L^{1} M^{11} Q^{3} T^{2}
246L	0	-	0	-
247P	0	-	0	-
248E	100	**D^{100}**	5	**D^{4}** N^{1}
249K	27.63	**Q^{24}** R^{1} T^{2}	1.2	**Q^{1}**
250D	0.02	N^{3} Q^{1}	4.8	E^{5}

	Computational Model *Neutral parameters*		Stanford Database *Pre-inhibitor Treatment*	
	% Non-Native	Predicted Mutations	% Non-Native	Observed Mutations
251S	99.87	N^{100}	1.6	I^{1}
252W	0	-	0	-
253T	0	-	0	-
254V	0.30	-	0	-
255N	69.73	D^{70}	0	-
256D	0.05	-	0	-
257I	99.71	**L^{100}**	0.7	**L^{1}**
258Q	0.74	L^{1}	0	-
259K	87.60	E^{66} N^{16} Q^{5}	0	-
260L	0	-	0	-
261V	80.77	I^{77} L^{4}	0.1	-
262G	100	A^{100}	0	-
263K	6.74	E^{3} Q^{3}	0	-
264L	0	-	0	-
265N	98.48	D^{98}	0	-
266W	5.86	L^{6}	0	-
267A	0	-	0	-
268S	0	-	0	-
269Q	1.94	L^{1} R^{1}	0	-
270I	0.13	-	0	-
271Y	3.44	F^{3}	0.2	-
272A	100	**P^{100}**	53.4	G^{1} **P^{45}** S^{6} T^{2}
273G	0	-	0	-
274I	4.87	L^{1} V^{3}	0.1	-
275K	1.83	**R^{1}** T^{1}	2.1	Q^{1} **R^{1}**
276V	100	D^{100}	4.7	I^{4} T^{1}
277K	99.90	E^{100}	40.7	R^{41}
278Q	92.41	**E^{92}**	5.1	**E^{3}** H^{2}
279L	0	-	0	-
280C	NA	NA	NA	NA
281K	0.13	-	5.7	R^{6}
282L	0	-	0	-
283L	0	-	6.2	I^{6}
284R	4.50	**K^{4}**	1.8	**K^{2}**
285G	0	-	0	-
286T	2.09	N^{2}	30.8	A^{29} P^{1}
287K	32.22	N^{2} Q^{13} T^{17}	0.2	-
288A	98.64	D^{22} P^{67} **S^{10}**	9.3	**S^{8}** T^{1}
289L	0	-	0	-
290T	0.03	-	0	-
291E	7.76	K^{8}	3.6	D^{4}
292V	3.56	**I^{3}**	9.5	**I^{10}**
293I	98.47	**V^{98}**	55.3	**V^{55}**
294P	0	-	10.6	A^{1} Q^{3} S^{2} T^{5}
295L	0.29	-	0.2	-
296T	3.44	**S^{3}**	1.2	**S^{1}**
297E	3.29	**K^{1}** **Q^{1}**	43.2	A^{14} **K^{19}** **Q^{1}** R^{4} T^{2} V^{2}
298E	0.87	-	0.6	A^{1}
299A	0	-	0	-
300E	16.09	Q^{16}	0.4	-

	Computational Model *Neutral parameters*		Stanford Database *Pre-inhibitor Treatment*	
	% Non-Native	Predicted Mutations	% Non-Native	Observed Mutations
301L	98.59	Q^{7} R^{91}	2	I^{2}
302E	0	-	0.3	-
303L	55.05	F^{45} I^{2} R^{8}	0	-
304A	54.33	D^{9} **E^{46}**	0.8	**E^{1}**
305E	0.09	-	0	-
306N	0	-	0	-
307R	3.56	K^{3}	0.1	-
308E	3.69	D^{3}	0	-
309I	0.01	-	0.2	-
310L	0	-	0.5	I^{1}
311K	10.60	E^{3} Q^{7}	8.7	R^{9}
312E	82.19	D^{41} G^{41}	7	K^{2} N^{1} Q^{1} T^{2} V^{1}
313P	0	-	1.6	Q^{1} T^{1}
314V	0.06	-	0.2	-
315H	100	D^{97} N^{2} Y^{1}	0.1	-
316G	0	-	0	-
317V	0.67	D^{1}	7.1	A^{7}
318Y	1.54	F^{2}	0	-
319Y	0.17	-	0.2	-
320D	0	-	0.2	-
321P	0	-	0.5	S^{1}
322S	1.53	**T^{2}**	9.9	A^{2} **T^{8}**
323K	0.35	-	0.1	-
324D	0.01	-	7.1	E^{6} P^{1}
325L	0	-	0.7	I^{1}
326I	38.50	F^{12} **V^{27}**	16.1	**V^{16}**
327A	0.68	S^{1}	1.6	V^{2}
328E	0	-	1	D^{1}
329I	0.02	-	23	L^{15} V^{8}
330Q	0.02	-	0	-
331K	0	-	0.1	-
332Q	3.60	L^{3}	0	-
333G	0	-	8.2	D^{1} E^{7}
334Q	0.69	-	32.8	D^{1} E^{6} H^{5} L^{16} N^{2} Y^{2}
335G	0	-	3.8	D^{3} S^{1}
336Q	1.85	E^{2}	0	-
337W	0	-	0	-
338T	0	-	2.7	S^{3}
339Y	0	-	0.2	-
340Q	99.33	R^{99}	0	-
341I	0	-	1.3	F^{1} V^{1}
342Y	0	-	1.1	F^{1}
343Q	0	-	0.1	-
344E	50.20	V^{50}	0.9	D^{1}
345P	0.02	-	2.8	Q^{2}
346F	31.04	S^{1} **Y^{30}**	5.4	H^{1} **Y^{5}**
347K	0.78	N^{1}	0.5	-
348N	34.09	D^{29} S^{5}	0.3	-
349L	0	-	0	-
350K	92.51	M^{91} R^{1}	0.4	-

	Computational Model *Neutral parameters*		Stanford Database *Pre-inhibitor Treatment*	
	% Non-Native	Predicted Mutations	% Non-Native	Observed Mutations
351T	0	-	0	-
352G	0	-	0	-
353K	99.33	T^{99}	0	-
354Y	0.02	-	0	-
355A	100	D^{89} **T^{7}** V^{3}	2.3	G^{1} **T^{1}**
356R	5.89	G^{6}	13.3	K^{13}
357M	100	**I^{5} K^{5} R^{63} T^{21} V^{5}**	30.1	A^{1} **I^{2} K^{3} R^{2} T^{20} V^{2}**
358R	99.62	G^{57} **K^{43}**	5.3	**K^{5}**
359G	0	-	8.8	S^{9}
360A	99.71	D^{1} S^{99}	14.4	T^{14} V^{1}
361H	99.81	D^{86} N^{7} Y^{7}	0	-
362T	4.53	N^{1} S^{3}	0	-
363N	0.23	-	0	-
364D	0	-	0.6	E^{1}
365V	0.08	-	3.3	I^{3}
366K	2.03	E^{1} **R^{1}**	10	**R^{10}**
367Q	92.42	E^{92}	0.6	L^{1}
368L	0	-	0	-
369T	7.59	**A^{8}**	6.2	**A^{4}** V^{1}
370E	0.01	-	1.9	D^{1}
371A	0.08	-	5.5	V^{5}
372V	0	-	0	-
373Q	9.78	E^{10}	0.6	H^{1}
374K	75.24	**R^{75}**	0.8	**R^{1}**
375I	0	-	6.3	V^{6}
376A	0.06	-	33.8	S^{6} T^{24} V^{3}
377T	99.87	**I^{58} K^{33} R^{8}**	17.2	**I^{2} K^{1}** L^{3} M^{4} Q^{4} **R^{1}** S^{1} V^{1}
378E	0	-	0	-
379S	0	-	6.9	G^{7}
380I	0.01	-	0.4	-
381V	0.06	-	4.4	**I^{4}**
382I	0.17	-	0.8	-
383W	0.06	-	0	-
384G	0	-	0	-
385K	64	**R^{63}** T^{1}	4.4	**R^{4}**
386T	1.22	P^{1}	18.7	A^{1} I^{16} V^{1}
387P	0	-	0	-
388K	0.02	-	1	R^{1}
389F	0	-	0	-
390K	0	-	52.2	R^{52}
391L	0	-	0.3	-
392P	0	-	0	-
393I	0	-	0.2	-
394Q	0.55	-	0.7	-
395K	1.32	**R^{1}**	3.9	**R^{4}**
396E	30.40	**D^{30}**	1.4	**D^{1}**
397T	0.39	-	0.5	-
398W	0	-	0	-
399E	69.81	Q^{69}	13.6	D^{14}
400A	99.93	D^{41} E^{1} **S^{18} T^{41}**	50	I^{2} L^{1} **S^{6} T^{40}**

	Computational Model *Neutral parameters*		Stanford Database *Pre-inhibitor Treatment*	
	% Non-Native	Predicted Mutations	% Non-Native	Observed Mutations
401W	0	-	0	-
402W	0	-	0	-
403T	98.48	K^{98}	24.7	A^{2} I^{6} L^{1} M^{12} S^{2} V^{1}
404E	12.59	**D^{3}** Q^{9}	11.6	**D^{12}**
405Y	0	-	1.2	H^{1}
406W	0	-	0	-
407Q	0.03	-	0	-
408A	0	-	0	-
409T	11.06	N^{6} S^{6}	0	-
410W	99.71	S^{99}	0	-
411I	3.45	**V^{3}**	1	**V^{1}**
412P	0	-	0	-
413E	1.54	**D^{2}**	0.9	**D^{1}**
414W	0	-	0	-
415E	1.55	V^{2}	0	-
416F	0	-	1	Y^{1}
417V	98.84	D^{99}	1.6	I^{1} T^{1}
418N	99.49	D^{99}	0.6	S^{1}
419T	25.27	I^{25} N^{1}	0	-
420P	0	-	0.2	-
421P	0	-	0.2	-
422L	0	-	0	-
423V	0	-	0	-
424K	99.54	N^{91} Q^{1} R^{4} T^{3}	0	-
425L	0	-	0	-
426W	0	-	0	-
427Y	0	-	0	-
428Q	1.66	E^{2}	0.2	-
429L	3.45	I^{3}	0	-
430E	69.71	Q^{70}	0	-
431K	47.46	E^{30} Q^{17}	4.9	R^{1} T^{3}
432E	7.62	Q^{8}	10.1	D^{10}
433P	0	-	0	-
434I	0.67	V^{1}	0.4	-
435V	100	D^{100}	40.1	A^{12} E^{7} I^{15} L^{3} M^{1} P^{1} T^{1}
436G	0	-	1.9	E^{2}
437A	99.33	S^{99}	2	V^{2}
438E	0	-	0	-
439T	0	-	0	-
440F	0	-	1.6	Y^{2}
441Y	0	-	0	-
442V	0	-	0	-
443D	100	G^{2} H^{98}	0	-
444G	0	-	0	-
445A	100	G^{84} S^{16}	0	-
446A	84.58	G^{82} **S^{3}**	5.5	**S^{6}**
447N	69.71	D^{70}	4.7	S^{4}
448R	7.59	**K^{8}**	0.7	**K^{1}**
449E	92.42	**D^{92}**	6.2	**D^{5}**
450T	96.07	N^{48} **S^{48}**	0.9	**S^{1}**

	Computational Model *Neutral parameters*		Stanford Database *Pre-inhibitor Treatment*	
	% Non-Native	Predicted Mutations	% Non-Native	Observed Mutations
451K	92.42	N^{92}	4.1	R^{4}
452L	0.40	-	14	A^{1} I^{5} K^{1} M^{1} Q^{1} S^{5} V^{1}
453G	0	-	0	-
454K	34.42	**R^{5}** T^{29}	4.8	**R^{5}**
455A	0.67	T^{1}	0	-
456G	0	-	0	-
457Y	0.02	-	0	-
458V	0.13	-	0.9	I^{1}
459T	0	-	0	-
460D	0.01	-	40	N^{38} S^{2}
461R	0.06	-	21.4	K^{21}
462G	0	-	0	-
463R	99.87	I^{100}	3.1	K^{3}
464Q	1.81	R^{2}	0.3	-
465K	7.59	R^{8}	0.4	-
466V	99.34	D^{97} G^{1} **I^{1}** L^{1}	6.8	A^{5} **I^{2}**
467V	0.92	D^{1}	13.8	I^{14}
468S	76.76	**P^{23}** **T^{53}**	32.6	H^{1} **P^{23}** **T^{9}**
469L	15.89	F^{16}	7.2	I^{6}
470T	0	-	12.8	A^{5} N^{4} P^{2} S^{1}
471D	100	G^{100}	6.3	E^{5} N^{1}
472T	0	-	0.3	-
473T	0.01	-	0	-
474N	99.07	D^{11} I^{5} K^{11} S^{60} T^{11}	0	-
475Q	31.18	E^{1} K^{30}	0	-
476K	8.14	T^{8}	2	Q^{2}
477T	96.56	**A^{96}**	4.6	**A^{4}**
478E	0	-	0	-
479L	0.03	-	0	-
480Q	0.67	L^{1}	6	E^{1} H^{5}
481A	0	-	0	-
482I	0.14	-	1	V^{1}
483H	100	**L^{70}** R^{30}	36.8	**L^{3}** N^{5} Q^{6} Y^{23}
484L	3.56	Q^{3}	0.3	-
485A	0	-	0	-
486L	0	-	0	-
487Q	0.06	-	0.7	-
488D	0	-	0	-
489S	0	-	0	-
490G	3.44	S^{3}	2.3	E^{2}
491L	96.56	**S^{97}**	30.6	A^{2} P^{2} **S^{22}** T^{1} V^{4}
492E	92.66	K^{89} Q^{3}	0.7	-
493V	84.20	A^{1} I^{84}	0	-
494N	0.39	-	0	-
495I	0	-	0.7	-
496V	0.01	-	0.3	-
497T	15.93	I^{16}	0.4	-
498D	0.01	-	0	-
499S	0	-	0	-
500Q	98.84	L^{99}	0	-

	Computational Model *Neutral parameters*		Stanford Database *Pre-inhibitor Treatment*	
	% Non-Native	Predicted Mutations	% Non-Native	Observed Mutations
501Y	51.04	F^{49} N^{2}	0	-
502A	50.04	**V^{50}**	1.7	**V^{1}**
503L	0.39	-	2.5	I^{2}
504G	100	W^{100}	0	-
505I	15.99	L^{16}	0	-
506I	84.13	**L^{84}**	0.9	**L^{1}**
507Q	50.60	L^{49} R^{1}	1.9	H^{2}
508A	100	D^{100}	0.3	-
509Q	0.29	-	2.7	H^{1} K^{1}
510P	0	-	0	-
511D	0.07		0	-
512K	100	E^{16} **T^{84}**	12.4	I^{1} Q^{4} R^{6} **T^{1}**
513S	0	-	0	-
514E	3.06	K^{2} Q^{2}	0.8	D^{1}
515S	70.04	L^{1} T^{69}	0	-
516E	31.07	**D^{30}** Q^{1}	1.9	**D^{1}**
517L	0.67	**I^{1}**	17	**I^{12}** V^{5}
518V	98.26	E^{49} L^{49}	0	-
519S	100	**N^{100}**	36	**N^{36}**
520Q	34.74	E^{1} **K^{28}** R^{5}	2.7	**K^{2}** L^{1}
521I	0	-	0	-
522I	0.13	-	0	-
523E	1.82	Q^{2}	0.3	-
524Q	96.81	**E^{7}** L^{89}	12.9	**E^{12}**
525L	0	-	0	-
526I	0.01	-	1.1	V^{1}
527K	0.69	-	13.9	N^{10} Q^{2} R^{2}
528K	7.59	T^{8}	0	-
529E	14.21	K^{7} Q^{7}	1.6	D^{2}
530K	7.59	**R^{8}**	4.3	**R^{4}**
531V	0.02	-	8	I^{8}
532Y	0	-	0	-
533L	96.56	F^{97}	0.4	-
534A	100	**S^{100}**	10.8	**S^{7}** T^{3}
535W	0	-	0	-
536V	99.96	F^{100}	0	-
537P	0	-	0	-
538A	100	P^{100}	0	-
539H	99.97	N^{100}	0	-
540K	100	N^{1} Q^{3} R^{3} T^{92}	0	-
541G	1.17	D^{1}	0	-
542I	25.11	F^{25}	0	-
543G	0	-	0	-
544G	0	-	0	-
545N	99.96	D^{1} H^{14} S^{43} T^{43}	0	-
546E	0	-	0.3	-
547Q	2.64	E^{3}	2	K^{2}
548V	99.61	A^{100}	4	I^{4}
549D	69.71	E^{70}	0	-
550K	98.38	N^{2} Q^{2} R^{34} T^{60}	0	-

	Computational Model *Neutral parameters*		Stanford Database *Pre-inhibitor Treatment*	
	% Non-Native	Predicted Mutations	% Non-Native	Observed Mutations
551L	0.01	-	0	-
552V	6.66	D^{3} I^{3}	0	-
553S	70.03	G^{1} N^{69}	0	-
554A	100	D^{100}	46.7	K^{1} N^{8} S^{18} T^{19}
555G	0.05	-	0	-
556I	100	N^{49} T^{49} **V^{2}**	2.7	**V^{3}**
557R	0.15	-	0	-
558K	99.79	E^{14} N^{14} T^{72}	13.3	R^{13}
559V	7.04	E^{1} N^{1} Q^{1} R^{1} T^{1}	8.3	I^{8}
560L	30.05	A^{4} D^{4} E^{4} F^{4} G^{4} I^{4} M^{4}	0	-
