# Supplementary material for: Prediction of Mutational Tolerance in HIV-1 Protease and Reverse Transcriptase Using Flexible Backbone Protein Design
Source: PLoS Comput Biol. 2012 Aug 23;8(8):e1002639. doi: 10.1371/journal.pcbi.1002639 (PMC3426558; doi:10.1371/journal.pcbi.1002639)
Supplement: Figure S5 — Predicted and observed HIV-1 reverse transcriptase amino acid substitutions for the selective model. Data format is as described in the legend to Figure S1. Residues not considered in the analysis of the predictions (black triangles) are described in Figure S9. (PDF) [file pcbi.1002639.s005.pdf]

Figure S5: Predicted and observed HIV-1 reverse transcriptase amino acid substitutions for the selective model

	**Computational Model** *Selective parameters*		**Stanford Database** *Post-inhibitor Treatment*	
	% Non-Native	Predicted Mutations	% Non-Native	Observed Mutations
1P	90.53	L^{16} Q^{50} R^{2} S^{16} T^{5}	0.1	-
2I	2.09	V^{2}	0.1	-
3S	0.22	-	0	-
4P	0	-	2.6	S^{2} T^{1}
5I	15.91	V^{16}	0	-
6E	84.49	D^{82} K^{1} Q^{2}	5.7	D^{4} K^{2}
7T	13.41	P^{12} S^{1}	0.6	-
8V	0.34		0.3	-
9P	1.86	Q^{2}	0	-
10V	0	-	0	-
11K	99.95	T^{100}	1.6	R^{2}
12L	0	-	0	-
13K	0.04	-	0.1	-
14P	0.06	-	0	-
15G	0	-	0	-
16M	99.14	K^{1} L^{24} T^{73}	0.5	V^{1}
17D	0.01		0	-
18G	0	-	0	-
19P	0	-	0	-
20K	82.34	M^{1} N^{54} Q^{1} R^{23} T^{3}	19.8	R^{20}
21V	0.43		1.4	I^{1}
22K	13.72	E^{3} N^{2} Q^{4} R^{1} T^{5}	0.6	R^{1}
23Q	0.01		0	-
24W	99.95	L^{85} R^{9} S^{5}	0	-
25P	0	-	0	-
26L	0	-	0	-
27T	3.37	P^{3}	1.2	S^{1}
28E	0.08	-	2.3	A^{1} K^{2}
29E	1.1	Q^{1}	0	-
30K	16.81	E^{1} Q^{16}	0	-
31I	0.13	-	0.8	L^{1}
32K	22.01	E^{2} N^{1} Q^{3} R^{17}	2.1	E^{1}
33A	0	-	0.1	-
34L	0	-	0.2	-
35V	95.22	E^{4} I^{15} L^{77}	30.1	I^{12} L^{4} M^{7} R^{1} T^{7}
36E	4.6	D^{1} K^{2} Q^{2}	0.6	-
37I	0.69	L^{1}	0.2	-
38C	NA	NA	NA	NA
39T	60.2	A^{1} K^{23} N^{26} R^{10}	19.7	A^{16} E^{2} K^{1} S^{1}
40E	7.83	K^{6} Q^{2}	2.5	D^{1} F^{2}
41M	15.89	L^{16}	54.4	L^{54}
42E	0.02	-	0.1	-
43K	34.89	E^{7} N^{21} Q^{2} R^{4}	21.5	E^{12} N^{3} Q^{6}
44E	45.87	D^{18} K^{10} Q^{18}	19.3	A^{3} D^{16}
45G	0	-	0	-
46K	15.9	R^{16}	0.2	-
47I	0.68	V^{1}	0	-
48S	1.16	T^{1}	2.2	T^{2}
49K	8.82	Q^{1} R^{2} T^{6}	4.9	R^{5}
50I	6.85	T^{3} V^{3}	0.7	V^{1}
51G	0	-	0	-
52P	0.6	T^{1}	0	-
53E	99.87	D^{100}	0.9	D^{1}
54N	99.93	D^{97} I^{3}	0.2	-
55P	0.13	-	0	-
56Y	0.05	-	0	-
57N	66.7	S^{67}	0	-
58T	1.6	I^{2}	0.3	-
59P	0	-	0	-
60V	1.17	I^{1}	15.4	I^{15}
61F	5.86	Y^{6}	0	-
62A	58.25	S^{14} T^{3} V^{42}	4.5	V^{5}
63I	0.74	V^{1}	0	-
64K	11.66	N^{10} R^{1} T^{1}	3.4	H^{1} R^{2}
65K	16.33	E^{9} N^{3} Q^{3} R^{1}	2.6	R^{3}
66K	100	N^{100}	0	-
67D	94.19	G^{41} N^{54}	50	E^{1} G^{3} N^{45}
68S	77.8	G^{51} N^{22} T^{4}	7.7	G^{7}
69T	99.33	N^{99}	21.8	D^{13} N^{7} S^{1}
70K	98.17	E^{17} Q^{51} T^{29}	23.7	G^{1} R^{22}
71W	0	-	0	-
72R	0.02		0	-
73K	99.99	E^{6} N^{91} Q^{2} T^{2}	0.1	-
74L	5.91	V^{6}	23.6	I^{8} V^{16}
75V	0	-	12.7	A^{1} I^{3} L^{1} M^{6} T^{3}
76D	0	-	0	-
77F	0	-	2.6	L^{3}
78R	0.01	-	0	-
79E	0	-	0.2	-
80L	0	-	0	-
81N	3.54	D^{3}	0	-
82K	5.39	N^{3} R^{2}	0.5	R^{1}
83R	0	-	9.4	K^{9}
84T	0	-	0	-
85Q	0.03	-	0	-
86D	0.59	-	0.3	-
87F	0	-	0	-
88W	91.42	S^{91}	0.1	-
89E	49.41	D^{4} K^{7} Q^{38}	0	-
90V	71.63	G^{65} I^{6}	3.7	I^{4}
91Q	1.76	L^{1}	0	-
92L	0.02	-	0	-
93G	0.03	-	0	-
94I	95.99	L^{21} T^{74}	0.6	L^{1}
95P	0	-	0	-
96H	0	-	0	-
97P	0	-	0	-
98A	99.89	S^{100}	15.2	G^{7} S^{8}
99G	0	-	0	-
100L	0	-	5.8	I^{6}

	Computational Model *Selective parameters*		Stanford Database *Post-inhibitor Treatment*	
	% Non-Native	Predicted Mutations	% Non-Native	Observed Mutations
101K	2.98	E^{1} T^{1}	15.5	E^{6} H^{2} P^{2} Q^{5} R^{1}
102K	32.26	E^{2} N^{22} Q^{7}	6.1	Q^{3} R^{2}
103K	11.82	Q^{3} R^{1} T^{8}	43	N^{40} R^{2} S^{2}
104K	6.07	N^{3} T^{2}	1.4	N^{1} R^{1}
105S	54.41	F^{35} Y^{20}	0.1	-
106V	0.01	-	5.2	A^{2} I^{3}
107T	0	-	0.2	-
108V	0	-	8.1	I^{8}
109L	0	-	0.8	I^{1}
110D	NA	NA	NA	NA
111V	84.14	I^{84}	1.6	I^{2}
112G	26.94	A^{3} S^{24}	0	-
113D	50.17	N^{50}	0.1	-
114A	0.01	-	0	-
115Y	0.23	-	2.3	F^{2}
116F	98.85	L^{98} S^{1}	3.1	Y^{3}
117S	8.22	L^{1} T^{6}	0.1	-
118V	0	-	32	I^{32}
119P	0	-	0	-
120L	0	-	0	-
121D	0.3	-	4.1	H^{2} Y^{2}
122K	35.57	E^{21} Q^{7} R^{7}	55.9	E^{53} P^{3}
123D	6.66	E^{3} N^{3}	28.3	E^{23} N^{4} S^{1}
124F	36.46	Y^{36}	0	-
125R	0	-	0	-
126K	1.92	E^{1}	0	-
127Y	15.9	F^{16}	0	-
128T	0	-	0	-
129A	0.02	-	0	-
130F	0	-	0	-
131T	0	-	0	-
132I	0.13	-	0.1	-
133P	0	-	0	-
134S	0.07	-	0	-
135I	68.88	K^{3} L^{21} R^{1} S^{12} T^{9} V^{24}	45	K^{1} L^{4} M^{2} R^{1} T^{30} V^{8}
136N	0.04	-	0	-
137N	0.71	H^{1}	0.1	-
138E	51.15	D^{49} Q^{2}	2.4	A^{2} Q^{1}
139T	0.14	-	1.7	K^{1}
140P	1.71	T^{1}	0	-
141G	0	-	0	-
142I	8.33	K^{2} L^{2} N^{1} T^{1} V^{3}	12.2	T^{2} V^{10}
143R	4.67	I^{3} M^{2}	0	-
144Y	0	-	0	-
145Q	75.31	R^{75}	0	-
146Y	1.16	F^{1}	0	-
147N	2.05	D^{2}	0	-
148V	0	-	0	-
149L	0	-	0	-
150P	0	-	0	-
151Q	95.51	P^{95}	3.9	M^{4}
152G	0	-	0	-
153W	0	-	0	-
154K	0	-	0	-
155G	0	-	0	-
156S	95.5	A^{96}	0	-
157P	4.97	A^{3} L^{1} T^{1}	0	-
158A	0.1	-	2.3	S^{2}
159I	0.01	-	0.1	-
160F	0.01	-	0	-
161Q	8.90	E^{6} L^{2} R^{1}	0	-
162S	30.40	N^{2} R^{5} T^{23}	12	A^{4} D^{3} N^{1} Y^{4}
163S	18.93	I^{1} N^{3} T^{15}	0.3	-
164M	99.78	L^{100}	0.1	-
165T	7.84	K^{4} N^{1} R^{2} S^{1}	2	I^{2}
166K	2.45	Q^{1} R^{1} T^{1}	7	R^{6}
167I	0.22	-	0.6	V^{1}
168L	0	-	0	-
169E	0.06	-	5.1	D^{5}
170P	91.53	A^{1} L^{3} Q^{78} R^{8} T^{2}	0	-
171F	0	-	0.4	-
172R	7.76	I^{6} K^{2}	1.6	K^{2}
173K	6.03	N^{2} Q^{3}	4.6	E^{1} N^{1} Q^{1} R^{1} T^{1}
174Q	5.01	E^{2} K^{2} R^{1}	6.9	E^{1} H^{1} K^{3} R^{2}
175N	16.02	H^{16}	1	Y^{1}
176P	0.92	-	0.1	-
177D	0.41	-	22.5	E^{20} G^{1} N^{1}
178I	15.98	V^{16}	14.4	L^{7} M^{7}
179V	0.52	-	12.6	D^{2} E^{1} I^{10}
180I	1.75	V^{2}	0.3	-
181Y	2.3	F^{2}	1.4	I^{1}
182Q	50.27	L^{50}	0	-
183Y	36.55	F^{36}	0	-
184M	0.89	R^{1}	53	I^{2} V^{51}
185D	NA	NA	NA	-
186D	NA	NA	NA	-
187L	0.02	-	0	-
188Y	50.00	F^{50}	6	L^{6}
189V	0	-	2.1	I^{2}
190G	1.28	A^{1}	19.5	A^{16} S^{3}
191S	0	-	0	-
192D	1.28	N^{1}	0.3	-
193L	9.78	S^{10}	0	-
194E	86.52	D^{41} K^{4} Q^{41}	0.4	-
195I	99.98	K^{27} L^{5} N^{5} R^{27} S^{9} T^{27}	1.2	L^{1}
196G	99.89	A^{1} D^{6} E^{90} R^{2} V^{1}	21.2	E^{20} K^{1}
197Q	28.83	E^{1} K^{13} L^{1} R^{13}	4.3	E^{2} K^{2}
198H	0	-	0	-
199R	0.05	-	0	-
200T	77.52	K^{68} N^{4} R^{4}	32	A^{24} E^{2} I^{4} K^{1}

	Computational Model *Selective parameters*		***Stanford Database*** *Post-inhibitor Treatment*	
	% Non-Native	Predicted Mutations	% Non-Native	Observed Mutations
201K	8.54	$N^{6}\ Q^{2}\ T^{1}$	0.1	-
202I	99.78	$N^{1}\ T^{1}\ V^{98}$	9.3	V^{9}
203E	3.19	$D^{2}\ Q^{1}$	16.4	$D^{6}\ K^{9}\ V^{1}$
204E	0.15	-	1.7	$D^{1}\ Q^{1}$
205L	0	-	0.1	-
206R	0.06	-	0	-
207Q	10.89	$E^{10}\ K^{1}\ R^{1}$	27.9	$A^{2}\ D^{2}\ E^{21}\ H^{1}\ K^{2}\ N^{1}$
208H	25.7	$D^{24}\ Q^{1}$	16.5	$F^{1}\ Y^{16}$
209L	0	-	0	-
210L	1.77	I^{1}	40.9	$F^{1}\ S^{1}\ W^{40}$
211R	2.01	K^{2}	59.8	$A^{1}\ D^{1}\ E^{1}\ G^{1}\ K^{53}\ Q^{1}\ S^{1}\ T^{1}$
212W	99.36	$G^{1}\ R^{95}\ S^{3}$	0.3	-
213G	0	-	0	-
214F	54.75	$I^{26}\ L^{26}\ V^{3}$	11.9	L^{12}
215T	5.39	$I^{1}\ K^{1}\ R^{3}$	66.4	$D^{1}\ F^{14}\ I^{1}\ V^{1}\ Y^{50}$
216T	0.29	-	0	-
217P	0	-	0	-
218D	0.05	-	10.9	E^{11}
219K	10	E^{10}	38.2	$D^{1}\ E^{8}\ N^{8}\ Q^{16}\ R^{6}$
220K	57.44	$E^{2}\ N^{43}\ Q^{5}\ T^{8}$	0	-
221H	99.91	$D^{24}\ N^{74}\ P^{2}$	5.4	Y^{5}
222Q	2.67	L^{3}	0	-
223K	99.61	T^{100}	9.2	$E^{4}\ Q^{4}\ T^{1}$
224E	99.34	$D^{56}\ G^{43}\ Q^{1}$	1.3	D^{1}
225P	0	-	2	H^{2}
226P	0.02	-	0	-
227F	97.99	Y^{98}	2.6	L^{2}
228L	50.4	$I^{9}\ Q^{28}\ R^{9}\ V^{3}$	22.8	$H^{17}\ R^{6}$
229W	0	-	0	-
230M	36.1	$K^{1}\ L^{21}\ R^{14}$	0.7	L^{1}
231G	0	-	0	-
232Y	0.67	F^{1}	0.1	-
233E	50.39	$D^{50}\ V^{1}$	0	-
234L	0.13	-	0.2	-
235H	0.95	Y^{1}	0	-
236P	0	-	0.2	-
237D	0.51	-	1.4	E^{1}
238K	66.67	$E^{1}\ N^{58}\ Q^{4}\ R^{4}\ T^{1}$	2.4	T^{2}
239W	0	-	0	-
240T	2.43	S^{2}	0	-
241V	96.56	D^{97}	0.3	-
242Q	0.72	E^{1}	2	H^{2}
243P	0.01	-	0.7	-
244I	2.14	V^{2}	0.4	-
245V	51.37	$D^{49}\ I^{1}\ L^{2}$	31.6	$E^{11}\ I^{1}\ K^{6}\ M^{6}\ Q^{3}\ R^{1}\ T^{4}$
246L	0.05	-	0	-
247P	0	-	0	-
248E	99.78	D^{100}	4.2	D^{3}
249K	36.34	$N^{2}\ Q^{28}\ R^{2}\ T^{5}$	0.8	Q^{1}
250D	0.22	$N^{6}\ Q^{2}\ T^{1}$	4.3	E^{4}

	Computational Model *Selective parameters*		***Stanford Database*** *Post-inhibitor Treatment*	
	% Non-Native	Predicted Mutations	% Non-Native	Observed Mutations
251S	98.03	$N^{96}\ T^{1}$	1.3	-
252W	0	-	0	-
253T	0.03	-	0	-
254V	1.19	A^{1}	0	-
255N	50.28	D^{50}	0	-
256D	0.38	-	0	-
257I	96.56	L^{97}	0.7	L^{1}
258Q	1.65	E^{1}	0	-
259K	85.56	$E^{50}\ N^{25}\ Q^{10}$	0	-
260L	0	-	0.2	-
261V	40.24	$I^{34}\ L^{6}$	0.1	-
262G	99.92	$A^{99}\ S^{1}$	0	-
263K	11.61	$E^{5}\ Q^{5}$	0.1	-
264L	0	-	0	-
265N	82.71	$D^{80}\ S^{1}\ T^{2}$	0	-
266W	6.74	L^{7}	0	-
267A	0.02	-	0	-
268S	0	-	0	-
269Q	3.84	$K^{1}\ L^{1}\ R^{2}$	0.1	-
270I	0.67	V^{1}	0	-
271Y	5.85	F^{6}	0	-
272A	100	$D^{1}\ P^{99}$	55.2	$P^{52}\ S^{3}$
273G	0	-	0	-
274I	8.92	$L^{3}\ V^{6}$	0.2	-
275K	6.07	$Q^{1}\ R^{3}\ T^{2}$	1.9	$Q^{1}\ R^{1}$
276V	99.98	D^{100}	2	$I^{1}\ T^{1}$
277K	94.99	$E^{93}\ Q^{2}\ T^{1}$	31.4	R^{31}
278Q	75.26	E^{75}	8.5	$E^{5}\ H^{4}$
279L	0.02	-	0.1	-
280C	NA	NA	NA	NA
281K	3.78	$E^{2}\ R^{1}$	5.5	R^{6}
282L	0	-	0	-
283L	0	-	7.7	I^{8}
284R	8.67	K^{9}	5	K^{5}
285G	0	-	0	-
286T	5.74	$N^{5}\ S^{1}$	31.5	$A^{30}\ P^{2}$
287K	35.3	$N^{4}\ Q^{12}\ T^{19}$	0	-
288A	89.15	$D^{12}\ E^{1}\ G^{3}\ P^{66}\ S^{7}$	10.8	$S^{9}\ T^{2}$
289L	0.04	-	0.1	-
290T	0.11	-	0	-
291E	10.8	$D^{1}\ K^{10}\ Q^{1}$	3.1	D^{3}
292V	6.46	$D^{1}\ I^{6}$	6.7	I^{7}
293I	90.23	V^{90}	53.9	V^{54}
294P	0.04	-	8.4	$A^{1}\ Q^{2}\ S^{1}\ T^{4}$
295L	1.16	P^{1}	0	-
296T	5.86	S^{6}	2.1	S^{2}
297E	7.73	$D^{1}\ K^{3}\ Q^{3}$	50	$A^{10}\ K^{29}\ Q^{2}\ R^{7}\ T^{1}$
298E	3.41	$D^{1}\ K^{1}\ Q^{1}$	0.6	-
299A	0.03	-	0	-
300E	16.72	$K^{1}\ Q^{16}$	0.1	-

	Computational Model *Selective parameters*		Stanford Database *Post-inhibitor Treatment*	
	% Non-Native	Predicted Mutations	% Non-Native	Observed Mutations
301L	91.72	I^{1} Q^{14} R^{76}	1.1	I^{1}
302E	0	-	0.6	D^{1}
303L	45.21	F^{31} I^{3} R^{10}	0.2	-
304A	43.41	D^{11} E^{32}	2.6	E^{1} G^{2}
305E	0.73	-	0.1	-
306N	0.01	-	0.1	-
307R	6.11	K^{6}	0.1	-
308E	7.08	D^{6} K^{1} Q^{1}	0.1	-
309I	0.16	-	0.2	-
310L	0	-	0.2	-
311K	15.07	E^{5} Q^{9}	6.1	R^{6}
312E	59.47	D^{41} G^{18} Q^{1}	2.3	Q^{1}
313P	0.04	-	1.1	S^{1}
314V	0.44	-	0.1	-
315H	99.88	D^{90} L^{1} N^{6} Q^{1} R^{1} Y^{1}	0.1	-
316G	0	-	0	-
317V	2.01	D^{2}	8.2	A^{8}
318Y	3.67	F^{3}	1.7	F^{2}
319Y	1.01	F^{1}	0.2	-
320D	0	-	0.1	-
321P	0.04	-	0.1	-
322S	3.45	T^{3}	7	A^{1} T^{6}
323K	1.55	Q^{1}	0.1	-
324D	0.11	-	12.1	E^{12}
325L	0.02	-	0.4	-
326I	34.77	F^{12} V^{21}	7.3	V^{7}
327A	2.13	S^{2}	0.9	V^{1}
328E	0.07	-	0.2	-
329I	0.22	-	18	L^{11} V^{7}
330Q	0.36	-	0	-
331K	0	-	0	-
332Q	1.83	E^{1} L^{1}	0	-
333G	0	-	9.7	D^{1} E^{9}
334Q	3.66	E^{1} P^{2}	34.4	D^{2} E^{8} H^{4} L^{13} N^{3} P^{1} R^{1} Y^{1}
335G	0	-	6	D^{2} S^{4}
336Q	4.67	E^{3} R^{1}	0	-
337W	0	-	0	-
338T	0	-	2	S^{2}
339Y	0.01	-	0.5	W^{1}
340Q	94.15	R^{94}	0	-
341I	0.02	-	1.2	F^{1}
342Y	0.01	-	0.7	F^{1}
343Q	0	-	0.1	-
344E	21.67	K^{1} Q^{2} V^{20}	0.8	D^{1}
345P	0.25	-	2.5	Q^{2}
346F	26.77	S^{3} Y^{24}	3.9	Y^{4}
347K	2.76	N^{2}	0.50	-
348N	30.62	D^{23} S^{8}	10.2	I^{10}
349L	0	-	0	-
350K	61.4	E^{1} M^{51} Q^{2} R^{4} T^{2}	2.7	R^{3}

	Computational Model *Selective parameters*		Stanford Database *Post-inhibitor Treatment*	
	% Non-Native	Predicted Mutations	% Non-Native	Observed Mutations
351T	0	-	0	-
352G	0	-	0	-
353K	94.32	E^{1} Q^{2} T^{91}	0	-
354Y	0.22	-	0	-
355A	99.98	D^{75} E^{2} S^{2} T^{14} V^{8}	1.5	G^{1}
356R	11.48	G^{11}	20.6	K^{21}
357M	99.98	I^{17} K^{9} L^{1} R^{23} T^{34} V^{17}	31.9	A^{1} I^{1} K^{1} R^{2} S^{1} T^{22} V^{3}
358R	99.36	G^{72} K^{27}	12.6	K^{13}
359G	0	-	30.7	S^{30}
360A	96.71	D^{3} S^{92} T^{1}	16	T^{11} V^{4}
361H	99.05	D^{81} N^{15} Y^{3}	0	-
362T	7.89	N^{2} S^{6}	0.1	-
363N	1.19	S^{1}	0	-
364D	0	-	0.4	-
365V	0.45	-	6.2	I^{6}
366K	3.86	E^{2} R^{1}	11.4	R^{11}
367Q	75.28	E^{75}	0.7	-
368L	0	-	0	-
369T	4.57	A^{4}	9	A^{3} V^{6}
370E	0.11	-	2.6	D^{2}
371A	0.61	-	27.2	T^{1} V^{26}
372V	0.01	-	0.3	-
373Q	7.6	E^{8}	0.5	H^{1}
374K	60.67	E^{1} R^{60}	0.4	-
375I	0.01	-	8.3	V^{8}
376A	0.45	-	27.3	S^{13} T^{12} V^{3}
377T	91.21	I^{41} K^{41} R^{10}	11.9	L^{4} M^{3} Q^{3} R^{2}
378E	0	-	0	-
379S	0	-	1.8	G^{2}
380I	0.11	-	0.1	-
381V	0.52	-	2	I^{2}
382I	1.07	L^{1}	0.6	-
383W	0.39	-	0	-
384G	0	-	0	-
385K	33.65	M^{1} N^{6} Q^{1} R^{11} T^{14}	1.5	R^{2}
386T	1.26	P^{1}	22.8	A^{5} I^{16} M^{1} V^{1}
387P	0	-	0	-
388K	0.19	-	0.5	R^{1}
389F	0.01	-	0	-
390K	0.01	-	45.8	R^{46}
391L	0	-	0	-
392P	0	-	0	-
393I	0.01	-	0.7	M^{1}
394Q	1.42	E^{1} K^{1}	1.8	L^{1}
395K	3.16	Q^{1} R^{2}	5.4	R^{5}
396E	40.86	D^{39} Q^{2}	1.7	D^{2}
397T	0.78	I^{1}	0.1	-
398W	0	-	0	-
399E	51	D^{1} K^{1} Q^{49}	17.7	D^{17} G^{1}
400A	95.49	D^{36} E^{2} S^{21} T^{36}	13.4	L^{1} S^{6} T^{6} V^{1}

	Computational Model *Selective parameters*		Stanford Database *Post-inhibitor Treatment*	
	% Non-Native	Predicted Mutations	% Non-Native	Observed Mutations
401W	0	-	0	-
402W	0.01	-	0	-
403T	90.49	K^{88} R^{3}	4.9	M^{5}
404E	18.5	D^{5} Q^{13}	7.8	D^{8}
405Y	0.07	-	0	-
406W	0	-	0	-
407Q	0.28	-	0	-
408A	0.2	-	0	-
409T	12.5	N^{6} S^{6}	0	-
410W	99.97	G^{5} S^{95}	0	-
411I	5.96	V^{6}	0	-
412P	0	-	0	-
413E	3.6	D^{3}	0	-
414W	0	-	0	-
415E	1.8	V^{2}	0	-
416F	0	-	0	-
417V	93.37	D^{93}	0	-
418N	92.41	D^{92}	0	-
419T	9.99	I^{8} N^{1}	0	-
420P	0	-	0	-
421P	0.05	-	0	-
422L	0	-	0	-
423V	0.2	-	0	-
424K	96.7	N^{81} Q^{5} R^{7} T^{4}	0	-
425L	0.08	-	0	-
426W	0.1	-	0	-
427Y	0	-	0	-
428Q	3.87	E^{3}	0	-
429L	5.88	I^{6}	0	-
430E	50.02	Q^{50}	0	-
431K	25.79	E^{9} Q^{16}	0	-
432E	18.1	Q^{18}	0	-
433P	0	-	0	-
434I	2.02	V^{2}	0	-
435V	99.87	D^{100}	14.9	A^{6} E^{2} I^{5} M^{2}
436G	0	-	0	-
437A	94.15	S^{94}	1.5	V^{2}
438E	0	-	0	-
439T	0	-	0	-
440F	0.07	-	0	-
441Y	0.01	-	0	-
442V	0	-	0	-
443D	99.88	G^{3} H^{97}	0	-
444G	0	-	0	-
445A	99.95	E^{1} G^{74} S^{25}	0	-
446A	65.89	G^{59} S^{6}	0	-
447N	50.01	D^{50}	0	-
448R	9.8	K^{10}	0	-
449E	75.46	D^{75} Q^{1}	0	-
450T	86.1	K^{2} N^{42} S^{42}	0	-

	Computational Model *Selective parameters*		Stanford Database *Post-inhibitor Treatment*	
	% Non-Native	Predicted Mutations	% Non-Native	Observed Mutations
451K	75.33	N^{75}	4.6	R^{5}
452L	2.2	I^{1} S^{1} V^{1}	0	-
453G	0	-	0	-
454K	31.3	I^{1} R^{7} T^{23}	0	-
455A	2.08	T^{2}	0	-
456G	0	-	0	-
457Y	0.22	-	0	-
458V	0.68	I^{1}	1.5	I^{2}
459T	0.01	-	0	-
460D	0.04	-	22.1	N^{22}
461R	0.42	-	8.5	K^{9}
462G	0	-	0	-
463R	95.7	I^{91} K^{4}	0	-
464Q	4.58	K^{1} R^{3}	0	-
465K	9.83	R^{10}	0	-
466V	94.7	D^{85} G^{3} I^{3} L^{3}	2.2	A^{2}
467V	3.31	D^{2} I^{1} L^{1}	3.1	I^{3}
468S	55.61	P^{11} T^{44}	6.1	P^{4} T^{2}
469L	15.98	F^{16}	3.1	I^{3}
470T	0.04	-	3	A^{2} P^{2}
471D	99.99	G^{100}	0	-
472T	0.01	-	0	-
473T	0.13	-	0	-
474N	95.03	D^{15} I^{4} K^{15} S^{46} T^{15}	0	-
475Q	27.36	E^{3} K^{24} R^{1}	0	-
476K	12.06	Q^{1} R^{1} T^{10}	0	-
477T	70.17	A^{69} R^{1}	0	-
478E	0.04	-	0	-
479L	0.29	-	0	-
480Q	0.93	L^{1}	1.5	H^{2}
481A	0.01	--	0	-
482I	0.89	L^{1}	0	-
483H	99.98	L^{43} R^{57}	6.4	N^{2} Y^{5}
484L	6.45	M^{1} Q^{6}	0	-
485A	0	-	0	-
486L	0	-	0	-
487Q	0.45	-	0	-
488D	0.01	-	0	-
489S	0	-	0	-
490G	5.86	S^{6}	0	-
491L	84.11	S^{84}	0	-
492E	77.11	K^{70} Q^{8}	0	-
493V	64.35	A^{2} I^{62}	0	-
494N	0.21	-	0	-
495I	0.01	-	0	-
496V	0.04	-	0	-
497T	7.92	I^{8}	0	-
498D	0.17	-	0	-
499S	0	-	0	-
500Q	16.6	L^{16}	0	-

	**Computational Model** *Selective parameters*		**Stanford Database** *Post-inhibitor Treatment*	
	% Non-Native	Predicted Mutations	% Non-Native	Observed Mutations
501Y	39.64	D^{1} F^{35} N^{4}	0	-
502A	36.82	V^{36}	0	-
503L	6.67	F^{7}	0	-
504G	100	A^{1} E^{17} R^{13} W^{69}	0	-
505I	16.52	L^{16} V^{1}	0	-
506I	63.75	L^{63}	0	-
507Q	26.03	E^{2} L^{18} R^{5}	0	-
508A	100	D^{99} E^{1}	0	-
509Q	1.2	K^{1}	0	-
510P	0	-	0	-
511D	0.51	-	0	-
512K	99.99	E^{25} T^{75}	0	-
513S	0.01	-	0	-
514E	7	K^{3} Q^{3}	0	-
515S	50.74	L^{1} T^{49}	0	-
516E	26.95	D^{24} Q^{3}	0	-
517L	2.03	I^{2}	0	-
518V	91.77	A^{2} D^{2} E^{44} I^{2} L^{44}	0	-
519S	99.96	N^{100}	2.4	N^{2}
520Q	32.17	E^{2} K^{22} R^{7}	0	-
521I	0	-	0	-
522I	0.67	L^{1}	0	-
523E	4.65	D^{1} Q^{3}	0	-
524Q	76.75	E^{23} L^{53}	0	-
525L	0	-	0	-
526I	0.18	-	0	-
527K	3.07	E^{1} Q^{1}	2.4	N^{2}
528K	9.8	T^{10}	0	-
529E	18.12	K^{9} Q^{9}	0	-
530K	9.78	R^{10}	0	-
531V	0.22	-	0	-
532Y	0.08	-	0	-
533L	84.18	F^{84}	0	-
534A	98.25	S^{98}	0	-
535W	0	-	0	-
536V	50	F^{50}	0	-
537P	0	-	0	-
538A	100	P^{99}	0	-
539H	99.5	D^{2} N^{98}	0	-
540K	99.96	N^{2} Q^{3} R^{3} T^{93}	0	-
541G	1.16	D^{1}	0	-
542I	5.72	F^{3} L^{2} V^{1}	0	-
543G	0.01	-	0	-
544G	0	-	0	-
545N	99.45	D^{2} H^{16} S^{41} T^{41}	0	-
546E	0.51	-	0	-
547Q	0.53	E^{1}	0	-
548V	97.37	A^{97}	0	-
549D	50.04	E^{50}	0	-
550K	92.99	N^{6} Q^{6} R^{24} T^{56}	0	-

	**Computational Model** *Selective parameters*		**Stanford Database** *Post-inhibitor Treatment*	
	% Non-Native	Predicted Mutations	% Non-Native	Observed Mutations
551L	0.10	-	0	-
552V	11.12	D^{6} I^{6}	0	-
553S	50.71	G^{1} N^{49}	0	-
554A	100	D^{99} S^{1}	3.7	T^{4}
555G	0.44	-	0	-
556I	99.90	L^{2} M^{2} N^{45} S^{2} T^{45} V^{5}	0	-
557R	0.39	-	0	-
558K	97.94	E^{19} N^{19} Q^{2} T^{58}	0	-
559V	14.01	E^{3} N^{3} Q^{3} R^{3} T^{3}	0	-
560L	30.05	A^{4} D^{4} E^{4} F^{4} G^{4} I^{4} M^{4}	0	-
