# Supplementary material for: Prediction of Mutational Tolerance in HIV-1 Protease and Reverse Transcriptase Using Flexible Backbone Protein Design
Source: PLoS Comput Biol. 2012 Aug 23;8(8):e1002639. doi: 10.1371/journal.pcbi.1002639 (PMC3426558; doi:10.1371/journal.pcbi.1002639)
Supplement: Table S5 — Predicted stabilization/destabilization effect of frequent major DRM combinations. Frequencies of HIV sequences that include mutation combinations with DRMs that are found in >50 sequences were obtained from the Stanford HIV database (http://hivdb.stanford.edu/pages/phenoSummary/Pheno.PI.Simple.html). The 1st and 2nd columns list the mutation combinations and sequence frequencies, respectively. Rosetta scores for these DRMs were assigned with wither “−" or “+" signs (for stabilizing (ERESFold<0) or destabilizing effects (ERESFold>0), respectively). DRMs for which our model did not provide predictions are given “?" signs. The 4th column is assigned with a positive sign in cases where a combination of predicted stabilizing and destabilizing mutations are found (corresponding to a compensation scenario). Negative signs are assigned where this is not the case (a “?" sign denotes the single case in which the compensating/non-compensating scenario could not be determined). (PDF) [file pcbi.1002639.s015.pdf]

Table S5: Predicted stabilization/destabilization effect of frequent major DRM combinations

Mutation combination	# sequences	Stabilizing/ destabilizing (Rosetta score)	combination of compensating mutations?
46I,90M	521	-,+	+
54V,82A,90M	420	+,+,+	-
46I,84V,90M	344	-,+,+	+
84V,90M	342	+,+	-
54V,82A	332	+,+	-
46I,54V,84V,90M	325	-,+,+,+	+
46L,54V,82A	314	-,+,+	+
46L,54V,82A,90M	299	-,+,+,+	+
46I,54V,82A	243	-,+,+	+
46I,54V,82A,90M	219	-,+,+,+	+
54V,84V,90M	144	+,+,+	-
54V,90M	116	+,+	-
30N,90M	108	-,+	+
46L,90M	94	-,+	+
46I,88S	88	-,+	+
30N,46I	86	-,-	-
46L,82A	86	-,+	+
82A,90M	79	+,+	-
54L,84V,90M	74	?,+,+	?
46I,84V	73	-,+	+
32I,46I,82A,90M	67	-,-,+,+	+
48V,54V,82A	67	+,+,+	-
46L,54V,84V,90M	62	-,+,+,+	+
46I,47V,54V,84V,90M	58	-,?,+,+,+	+
32I,46I,47V,54M,82A,90M	57	-,-,?,+,+,+	+
46I,54V,82F,90M	57	-,+,+,+	+
48V,54T,82A	57	+,+,+	-
46L,54V,82A,84V,90M	56	-,+,+,+,+	+
46L,54V,82A,84V	56	-,+,+,+	+
46I,54V,76V,82A	55	-,+,+,+	+
54V,82A,84V,90M	53	+,+,+,+	-
46I,50V,54V,82A,90M	52	-,?,+,+,+	+
48V,54V,82A,90M	52	+,+,+,+	-
46I,54V,84V	51	-,+,+	+
