# Supplementary material for: Prediction of Mutational Tolerance in HIV-1 Protease and Reverse Transcriptase Using Flexible Backbone Protein Design
Source: PLoS Comput Biol. 2012 Aug 23;8(8):e1002639. doi: 10.1371/journal.pcbi.1002639 (PMC3426558; doi:10.1371/journal.pcbi.1002639)
Supplement: Text S1 — Additional details of computational methods. Description of the Rosetta energy terms, the command line for Rosetta backrub simulations, computational time requirements and simulations to estimate the effect of correlated mutations. (PDF) [file pcbi.1002639.s016.pdf]

Text S1

Rosetta energy terms used in energy calculations

The contribution towards fold stability ($ERES_{Fold}$) of each mutated residue i at each sequence position j was estimated by recording the sum of inter- and intra-residue Rosetta energy terms:

$ERES_{Fold,\ i,j}$ = E_LJ_{atr} ($_{i,j}$) + E_LJ_{rep} ($_{i,j}$) + G_sol ($_{i,j}$) + E_dun ($_{i,j}$) + E_hb ($_{i,j}$) + E_prob ($_{i,j}$) + E_pair ($_{i,j}$) – E_ref ($_{i,j}$), where:

E_LJ_{atr}: attractive component of Rosetta Lennard-Jones potential

E_LJ_{rep}: repulsive component of Rosetta Lennard-Jones potential

G_sol: Rosetta implicit solvation term

E_dun: Rosetta rotamer probability term

E_hb: Rosetta hydrogen bonding term

E_prob: Rosetta probability of amino acid type, given the backbone conformation

E_pair: Rosetta pair term

E_ref: Rosetta reference energy

The contribution of each mutated residue to stability of the dimer interface ($ERES_{Dimer}$) was estimated by calculating only inter-chain pair-wise Rosetta energy function contributions between the mutated residue and neighboring residues on the opposite dimer chain (each interface term was calculated only over sets of residues where chain(residue 1) != chain (residue2)):

$ERES_{Dimer}$ ($_{i,j}$) = E_LJ_{atr}_interface ($_{i,j}$) + E_LJ_{rep}_interface ($_{i,j}$) + G_sol_interface ($_{i,j}$) + E_hb_interface ($_{i,j}$) + E_pair_interface ($_{i,j}$)

Rosetta Backrub simulations

Backrub simulations used Rosetta svn version 15373 and the following options: rosetta.gcc -backrub_mc -fa_input -s [starting structure] -use_pdb_numbering -read_all_chains chain _ -find_disulf -resfile [resfile] -ex1 -ex2 -skip_missing_residues -extrachi_cutoff 0 -ntrials 10000 -

max_res 12 -only_bb .5 -only_rot .5 -nstruct 100

Computational time

Running time for generating all mutations per single structure was 22 hours for reverse transcriptase and 2¾ hours for protease. Generation of one backrub structure was 28 minutes for reverse transcriptase and 6 minutes for protease (AMD Opteron 275; 2.2GHz).

Estimating the effect of correlated mutations for a finite set of double mutations

Rosetta was used to model each of a set of initial single mutation (10I, 13V, 15V, 30N, 35D, 36I, 46I, 54V, 62V, 63P, 64V, 71V, 77I, 82A, 84V, 90M and 93L, selected to occur frequently in the Stanford database) onto each of the 263 experimentally determined protease structures (which had been reverted to the consensus HIV-1 protease sequence, as described in the main manuscript). Neighboring side-chains were allowed to repack during mutation and the resulting structures were subjected to another round of side-chain minimization.

Each ensemble of mutated structures containing one of the single initial mutations was then used to predict the mutational frequencies for all secondary mutations (19 amino acid types at each protease site), as described for the original model. Finally, mutational frequencies predicted in the presence of each single initial mutation were compared to the mutational frequencies predicted in the absence of a prior mutation from the consensus sequence. This simple model does not account for the energetic effect of the initial mutations, *i.e.* mutations in the background of an initial stabilizing mutation are not more likely to be tolerated than mutations in the background of an initial destabilizing mutation.

Figure S7 lists all protease mutations that showed an appreciable change in predicted mutational frequency towards that observed in the Stanford database under protease inhibitor treatment when an initial mutation was present. The tolerance to the same mutation was often observed to increase in the presence of more than one individual initial mutation. For example,

the frequency of mutation 20R was moderately increased in 4 separate initial mutation simulations while the mutation 72V was slightly increased in 5 initial mutation simulations. This suggests that some mutations may have several separate paths to mutational tolerance. The majority of observed changes in mutational frequencies were modest (1%-3%), although the double mutations 20R-36I, 62V-13V, 62V-54V, 71V-64V and 77I-64V were observed to have particularly large predicted tolerance changes (>10%). The double mutation analysis was only done for a small set of initial protease mutations. Nevertheless, it resulted in the recapitulation of tolerance for seven mutations occurring within the protease mutation database under inhibitor treatment at low levels (20I, 23I, 33F/I, 53L, 64M and 75I) that were not predicted by the original model (**Figure S7**).
